# Characteristics of the sources, evaluation, and grading of the certainty of evidence in systematic reviews in public health: A methodological study

**DOI:** 10.3389/fpubh.2023.998588

**Published:** 2023-03-30

**Authors:** Yangqin Xun, Qiangqiang Guo, Mengjuan Ren, Yunlan Liu, Yajia Sun, Shouyuan Wu, Hui Lan, Juanjuan Zhang, Hui Liu, Jianjian Wang, Qianling Shi, Qi Wang, Ping Wang, Yaolong Chen, Ruitai Shao, Dong Roman Xu

**Affiliations:** ^1^Evidence-Based Medicine Center, School of Basic Medical Sciences, Lanzhou University, Lanzhou, China; ^2^School of Public Health, Lanzhou University, Lanzhou, China; ^3^West China School of Public Health and West China Fourth Hospital, Sichuan University, Chengdu, China; ^4^The First School of Clinical Medicine, Lanzhou University, Lanzhou, China; ^5^Department of Health Research Methods, Evidence and Impact, Faculty of Health Sciences, McMaster University, Hamilton, ON, Canada; ^6^McMaster Health Forum, McMaster University, Hamilton, ON, Canada; ^7^Research Unit of Evidence-Based Evaluation and Guidelines, Chinese Academy of Medical Sciences (2021RU017), School of Basic Medical Sciences, Lanzhou University, Lanzhou, China; ^8^Lanzhou University, An Affiliate of the Cochrane China Network, Lanzhou, China; ^9^World Health Organization (WHO) Collaborating Centre for Guideline Implementation and Knowledge Translation, Lanzhou, China; ^10^School of Population Medicine and Public Health, Chinese Academy of Medical Sciences & Peking Union Medical College, Beijing, China; ^11^Department of Non-communicable Diseases, World Health Organization (WHO), Geneva, Switzerland; ^12^SMU Institute for Global Health (SIGHT), School of Health Management and Dermatology Hospital, Southern Medical University (SMU), Guangzhou, China

**Keywords:** public health, evidence, quality assessment, certainty of evidence, systematic reviews, methodological survey

## Abstract

**Objectives:**

To systematically explore how the sources of evidence, types of primary studies, and tools used to assess the quality of the primary studies vary across systematic reviews (SRs) in public health.

**Methods:**

We conducted a methodological survey of SRs in public health by searching the of literature in selected journals from electronic bibliographic databases. We selected a 10% random sample of the SRs that met the explicit inclusion criteria. Two researchers independently extracted data for analysis.

**Results:**

We selected 301 SRs for analysis: 94 (31.2%) of these were pre-registered, and 211 (70.1%) declared to have followed published reporting standard. All SRs searched for evidence in electronic bibliographic databases, and more than half (*n* = 180, 60.0%) searched also the references of the included studies. The common types of primary studies included in the SRs were primarily cross-sectional studies (*n* = 132, 43.8%), cohort studies (*n* = 126, 41.9%), randomized controlled trials (RCTs, *n* = 89, 29.6%), quasi-experimental studies (*n* = 83, 27.6%), case-control studies (*n* = 58, 19.3%) qualitative studies (*n* = 38, 12.6%) and mixed-methods studies (*n* = 32, 10.6%). The most frequently used quality assessment tools were the Newcastle-Ottawa Scale (used for 50.0% of cohort studies and 55.6% of case-control studies), Cochrane Collaboration's Risk of Bias tool (50.7% of RCTs) and Critical Appraisal Skills Program (38.5% of qualitative studies). Only 20 (6.6%) of the SRs assessed the certainty of the body of evidence, of which 19 (95.0%) used the GRADE approach. More than 65% of the evidence in the SRs using GRADE was of low or very low certainty.

**Conclusions:**

SRs should always assess the quality both at the individual study level and the body of evidence for outcomes, which will benefit patients, health care practitioners, and policymakers.

## 1. Introduction

The term *Evidence-Based Medicine* (EBM) was first used in the scientific literature in 1991 (1). After 30 years, the concepts and methods of EBM have gradually penetrated into other research fields and subjects, including public health. Evidence-Based Public Health (EBPH) aims to integrate science-based interventions with the actual national and regional needs and priorities to improve the health of the population (2–4).

Public health professionals should always review the existing scientific evidence when planning and implementing projects, developing policies, and assessing progress (5). EBPH involves the systematic and comprehensive identification and evaluation of the best available evidence, to provide an explicit and valid scientific basis for public health policy making. However, studies have shown that the evidence in the area of public health is often insufficient to support decision-making, and the methodological approaches and quality of the evidence vary widely (6–8). Effective decision-making in public health requires high-quality evidence (9). High risk of bias in research evidence reduces the overall certainty of the body of evidence, which can lead to tentative or conditional recommendations, and potentially even to decisions that are harmful to patients and populations. It is therefore important to assess both the risk of bias in individual studies and the overall quality (or certainty) of the body of the evidence used for decision-making.

Systematic reviews (SRs) are now widely used to inform public health policies (10) and decisions (11). Evidence retrieval, evaluation, and quality assessment are key steps when conducting SRs. Researchers have explored how these steps were completed in SRs on certain topics, for instance, biomedical investigations (12), preclinical studies (13) and nutritional epidemiologic studies (14, 15). Many problems and limitations were found in these areas.

Given the specific and complex nature of public health as a field of research, a methodological investigation of systematic evaluation of evidence in public health research is needed. To date, no study has however assessed how these essential steps of evidence collection and synthesis are executed in SRs on public health topics. Therefore, this study aims to investigate how the sources, types and quality assessment methods of evidence vary across SRs in public health. The results of this study will provide valuable insights for systematic reviewers, journal editors, primary researchers, public health professionals, and policy-makers on the identification and assessment methods of primary studies and the distribution of different study types among them and how systematic review teams in the field of public health should improve their work.

## 2. Materials and methods

### 2.1. Study design

We performed a methodological survey of SRs in public health, randomly sampled from publications identified through a comprehensive literature review.

### 2.2. Study selection

We used the filter category “Public, Environmental & Occupational Health” in the “Journal Citation Reports” module of the Web of Science (16) to limit the number of journals. We also ran a supplementary manual search of ten English medical journals that had the terms “evidence-based” and “systematic review” in their title. Then, limited to the journals mentioned above, the search strategy using the string “meta-analysis” OR “systematic review” OR “systematic assessment” OR “integrative review” OR “research synthesis” OR “research integration” in the title was constructed. We applied this search strategy from January 2018 to April 2021 to Medline (*via* PubMed). We present the details of the search strategy in Appendix 1. Two reviewers conducted the electronic database search independently and discussed the results until a consensus was reached.

### 2.3. Eligibility criteria

#### 2.3.1. Inclusion criteria

We included SRs in public health that met the Preferred Reporting Items for Systematic Reviews and Meta-Analysis Protocols (PRISMA-P) definition of a systematic review, that is, articles that explicitly state the methods of study identification (i.e., a search strategy), study selection (e.g., eligibility criteria and selection process), and synthesis (or other types of summary) (17). Public health is defined as the promotion and protection health and wellbeing, prevention of illness and prolongation of life through organized societal efforts, and it includes three key domains: health improvement, improving services, and health protection (18). We included only published SRs written in English.

#### 2.3.2. Exclusion criteria

We excluded the following types of articles: systematic review of guidelines; overviews of reviews (or umbrella reviews); scoping reviews; methodological studies that included a systematic search for studies to evaluate some aspect of conduct or reporting; and protocols or summaries of SRs. We excluded articles for which the full text was not accessible. We also excluded commentaries, editorials, letters, summaries, conference papers and abstracts.

### 2.4. Literature screening and data extraction

Literature screening and data extraction were conducted by two researchers (eight researchers divided into four pairs) independently. Disagreements were resolved by discussion or consultation with a third researcher until consensus was reached. The titles and abstracts were first screened and obviously irrelevant publications were excluded. Then the full texts of the potentially eligible articles were read to determine if the article met the inclusion criteria. Finally, the included SRs were randomly ordered and a 10% sample was randomly selected using the RAND function in Microsoft Excel 2010 (Redmond, WA, USA). In this article, we present the flow diagram of the literature search, inclusion and exclusion criteria using the PRISMA 2020 27-item checklist (19).

Data were extracted from the final study sample using a standard data extraction form by two researchers independently. The data extraction form was developed through two rounds of pre-test followed by and discussions. The form included basic information (year of publication, journal of publication, country or region of the first author, number of authors, platform of registration, reporting statement, and funding); the name and number of included databases, websites, registration platforms and other supplementary search sources; the types and numbers of studies included in the SR (the study type is directly extracted according to the type reported in the SR); quality assessment tools used for included studies included in the SR; and the approach used to assess the quality (or certainty) of the body of evidence.

### 2.5. Statistical analysis

The tools used for quality assessment of the individual studies in each SR were identified from the full texts of each study report. The Newcastle-Ottawa Scale (NOS) tool is commonly used for cohort studies and case-control studies, the Cochrane Collaboration's Risk of Bias (ROB) tool for randomized controlled trials (RCTs), and the Critical Appraisal Skills Program (CASP) tool for qualitative studies. In these all three tools, each item is assigned a value 1 if the answer to the corresponding question was “yes” or “partial yes”, and 0 if the answer was “no” or “cannot tell”. Specially, the “Comparability” item in NOS may get a maximum value of 2, as it assesses the study controls for the most important factor and a second important factor. The risk of bias is defined as low if the total score for each primary study is ≥70% of the possible maximum score; high if the total score was ≤ 35% of the possible maximum score; and moderate if the total score was between 35 and 70% of the possible maximum score. We calculated on the item scores directly from the assessment of the authors of the include reviews, and then evaluated the overall methodological quality of the primary studies as described above.

We performed a descriptive analysis of the basic characteristics and reporting features of the included SRs. Continuous variables were expressed as medians and interquartile ranges (IQR), and categorical variables frequencies and percentages. We performed all statistical analyses using IBM SPSS 26.0 (Armonk, NY, USA).

## 3. Results

**Figure 1:**
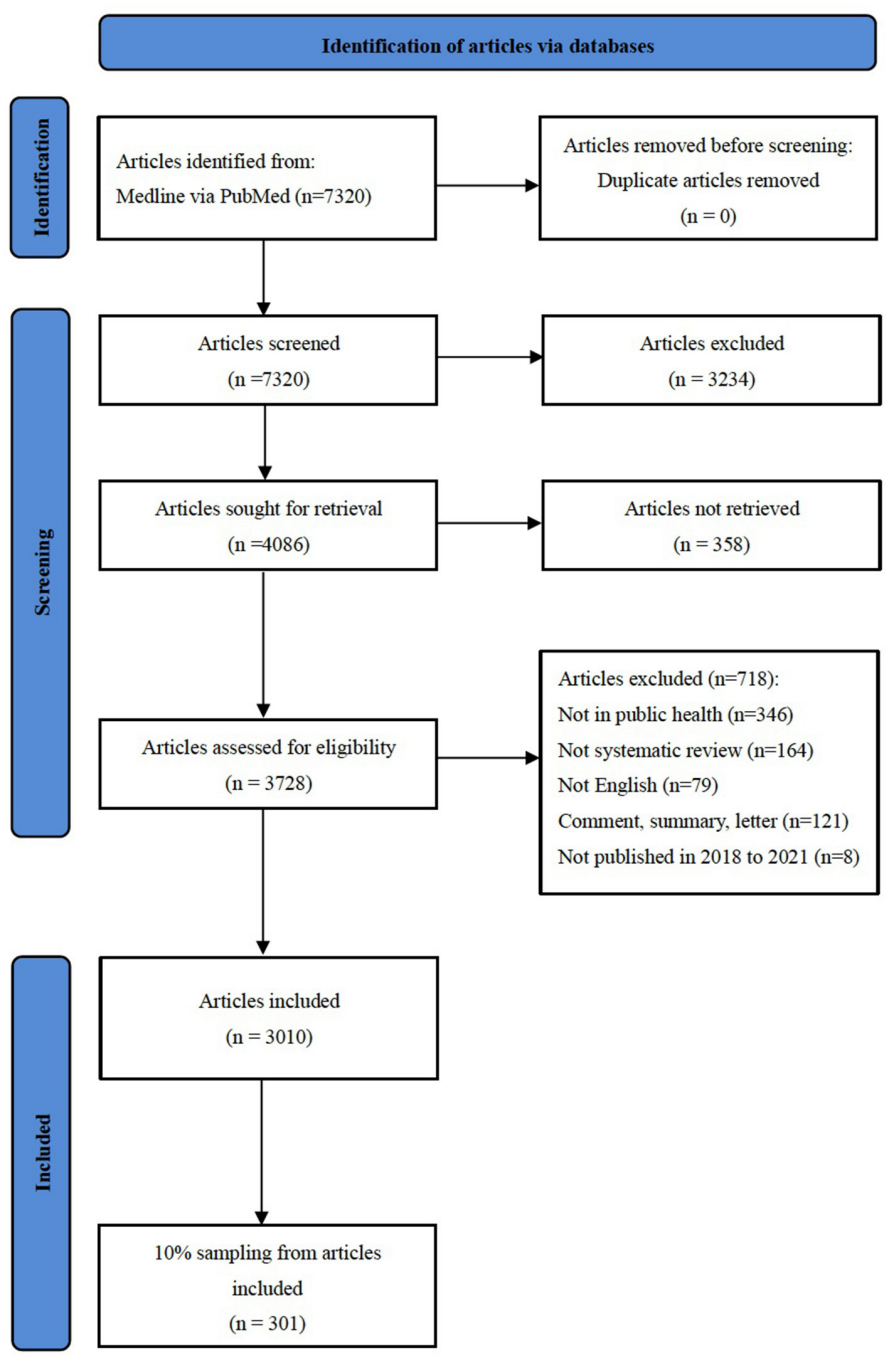
Flow diagram of the literature search.

The initial search yielded 7,320 articles. After screening titles, abstracts, 3,010 articles were retrieved, and 10% of the retrieved articles (*n* = 301) were randomly selected for inclusion (Figure 1).

### 3.1. Characteristics of the included studies

Characteristics of the included studies are shown in Table 1. The number of published SRs in public health increased over time. The first authors came from a total of 42 countries. The median number of authors per SR was five (IQR 4–6), and four (1.3%) SRs were conducted by one author. The median number of included studies was 19 (IQR 12–35), and only one (0.3%) SR identified no eligible studies. The SRs were published in 116 different journals among which the *International Journal of Environmental Research and Public Health* had the highest number of publications (*n* = 5,217.3%). Only one-third of the SRs were registered (*n* = 94, 31.2%). However, more than half of the SRs declared to have followed reporting guidances (*n* = 211, 70.1%). Almost three quarters of the SRs (*n* = 221, 73.4%) evaluated the methodological quality of the primary studies included in the SRs, however, only 6.6% (*n* = 20) of the SRs assessed the certainty of the body of evidence. Half of the SRs (*n* = 146, 48.5%) reported the funding sources for the SR. For about one fourth of the SRs (*n* = 81, 27.0%) were classified as etiology, for 69 (23.0%) as distribution, for 46 (15.3%) as therapeutic, for 40 (13.3%) as prevention, for eight (2.6%) as screening, for four (1.3%) as diagnosis or prognosis, and for 53 (17.5%) as other.

**Table 1:** Characteristics of the included systematic reviews (*n* = 301).

**Variables**	***n* (%)**
**Year of publication**	
2018	60 (20.0)
2019	87 (28.9)
2020	119 (39.5)
2021	35 (11.6)
Number of authors (Median, IQR)	5 (4–6)
**Country of the first author**	
United States of America	56 (18.6)
United Kingdom	35 (11.6)
Australia	30 (10.0)
China	30 (10.0)
Iran	14 (4.7)
Canada	13 (4.3)
Spain	13 (4.3)
Germany	12 (4.0)
Ethiopia	9 (3.0)
Italy	9 (3.0)
Korea	9 (3.0)
Netherlands	8 (2.6)
Brazil	6 (2.0)
Switzerland	6 (2.0)
Japan	5 (1.7)
Others^a^	46 (15.2)
**Protocol registration**	
PROSPERO	92 (30.6)
Open Science Framework	2 (0.6)
None	207 (68.8)
**Reporting statements cited** ^b^	
PRISMA and its extensions	204 (67.8)
MOOSE	10 (3.3)
ENTREQ	2 (0.7)
Others^c^	3 (1.0)
No	90 (30.0)
**Funding for the systematic review reported**	
Yes	146 (48.5)
No	155 (51.5)
**Methodological quality of the primary studies assessed**	
Yes	221 (73.4)
No	80 (26.6)
**Quality of the body of evidence assessed**	
Yes	20 (6.6)
Etiology	81 (27.0)
Distribution	69 (23.0)
Therapeutic	46 (15.3)
Prevention	40 (13.3)
Screening	8 (2.6)
Diagnosis/Prognosis	4 (1.3)
Other	53 (17.5)
**Systematic reviews of interventions**	
Yes	86 (28.6)
No	215 (71.4)
Number of the included primary studies (Median, IQR)	19 (12–35)

a Others included France (*n* = 4), Greece (*n* = 4), Portugal (*n* = 4), Singapore (*n* = 3), Thailand (*n* = 3), Belgium (*n* = 2), Cameroon (*n* = 2), Finland (*n* = 2), Norway (n =2), South Africa (*n* = 2), United Arab Emirates (*n* = 2), Austria (*n* = 1), Bangladesh (*n* = 1), Chile (*n* = 1), Colombia (*n* = 1), Denmark (*n* = 1), Ireland (*n* = 1), Lebanon (*n* = 1), Malaysia (*n* = 1), New Zealand (*n* = 1), Nigeria (*n* = 1), Pakistan (*n* = 1), Philippines (*n* = 1), Serbia (*n* = 1), Slovenia (*n* = 1), Sweden (*n* = 1), Tanzania (*n* = 1).

b Some studies reported following two or more reporting statements.

c Others included the Center for Reviews and Dissemination, Method for the thematic synthesis of qualitative research in systematic reviews, ROSES (RepOrting Standards for Systematic Evidence Syntheses).

ENTREQ, Enhancing transparency in reporting the synthesis of qualitative research; IQR, interquartile range; MOOSE, Meta-analysis Of Observational Studies in Epidemiology; PRISMA, Preferred Reporting Items for Systematic Reviews and Meta-Analyses.

### 3.2. Sources of evidence

All SRs included in this study searched electronic bibliographic databases for evidence (Table 2). A total of 173 different databases were searched, the total frequency was 1,331, and the median number of databases retrieved per SR was four (IQR, 3–5). Some database weresearched through various platforms varied for some of the databases. For example, Medline was accessed through EBSCO, Web of Science, Ovid, and the Virtual Health Library. Sixty percent of the SRs (*n* = 180) searched the references of included studies in addition to electronic databases. A total of 62 websites [the total frequency was 80, and the most commonly searched for websites was the World Health Organization (*n* = 5)] and 8 registration platforms [the total frequency was 55, and the most commonly searched was the Cochrane Central Register of Controlled Trials (*n* = 28)].

**Table 2:** Sources of primary studies most frequently searched in the included systematic reviews (*n* = 301).

**No**.	**Databases**	***n* (%)**
1	PubMed	181 (60.1)
2	Medline	161 (53.5)
3	Embase	159 (52.8)
4	Web of science	126 (41.9)
5	PsycInfo	94 (31.2)
6	CINAHL (Cumulative Index to Nursing and Allied Health Literature)	93 (30.9)
7	Scopus	77 (25.6)
8	Cochrane library	64 (21.3)
9	Science direct	22 (7.3)
10	CNKI (Chinese National Knowledge Infrastructure)	12 (4.0)
11	SPORTDiscus	12 (4.0)
12	ERIC (Educational Resources Information Center)	11 (3.6)
13	LILACS (Latin American and Caribbean Health Sciences Literature)	11 (3.6)
14	EBSCO	10 (3.3)
15	Global health	10 (3.3)
**No**.	**Websites**	**n (%)**
1	WHO (World Health Organization)	5 (1.7)
2	NICE (National Institute for Health and Care Excellence)	3 (1.0)
3	UNICEF (United Nations Children's Fund)	3 (1.0)
4	US-CDC (United States Centers for Disease Control and Prevention)	3 (1.0)
5	ECDC (European Center for Disease Prevention and Control)	2 (0.7)
6	ICRC (International Committee of the Red Cross)	2 (0.7)
7	IOM (International Organization of Migration)	2 (0.7)
8	IRC (International Rescue Committee)	2 (0.7)
9	MSF (Médecins Sans Frontières)	2 (0.7)
10	ReliefWeb	2 (0.7)
11	UNAIDS (Joint United Nations Programme on HIV/AIDS)	2 (0.7)
12	UNHCR (United Nations High Commissioner for Refugees)	2 (0.7)
**No**.	**Registration platforms**	***n*** **(%)**
1	CENTRAL (Cochrane Central Register of Controlled Trials)	28 (9.3)
2	ClinicalTrials.gov	13 (4.3)
3	ICTRP (WHO International Clinical Trials Registry Platform)	8 (2.6)
4	Australian New Zealand Clinical Trials Registry	2 (0.7)
**No**.	**Other sources**	***n*** **(%)**
1	Scanning the reference lists and other relevant review articles	180 (60.0)
2	Google Scholar	63 (20.9)
3	Google	15 (5.0)
4	Experts' consultation	12 (4.0)
5	Conference proceedings	11 (3.6)
6	Contact the authors	10 (3.3)
7	Specialist journals	7 (2.3)
8	Other handing searching methods	23 (7.6)

Some researchers regard PubMed as a database rather than a search platform. And we only extracted data that were reported in each SR.

### 3.3. Types of primary studies of SRs

**Figure 2:**
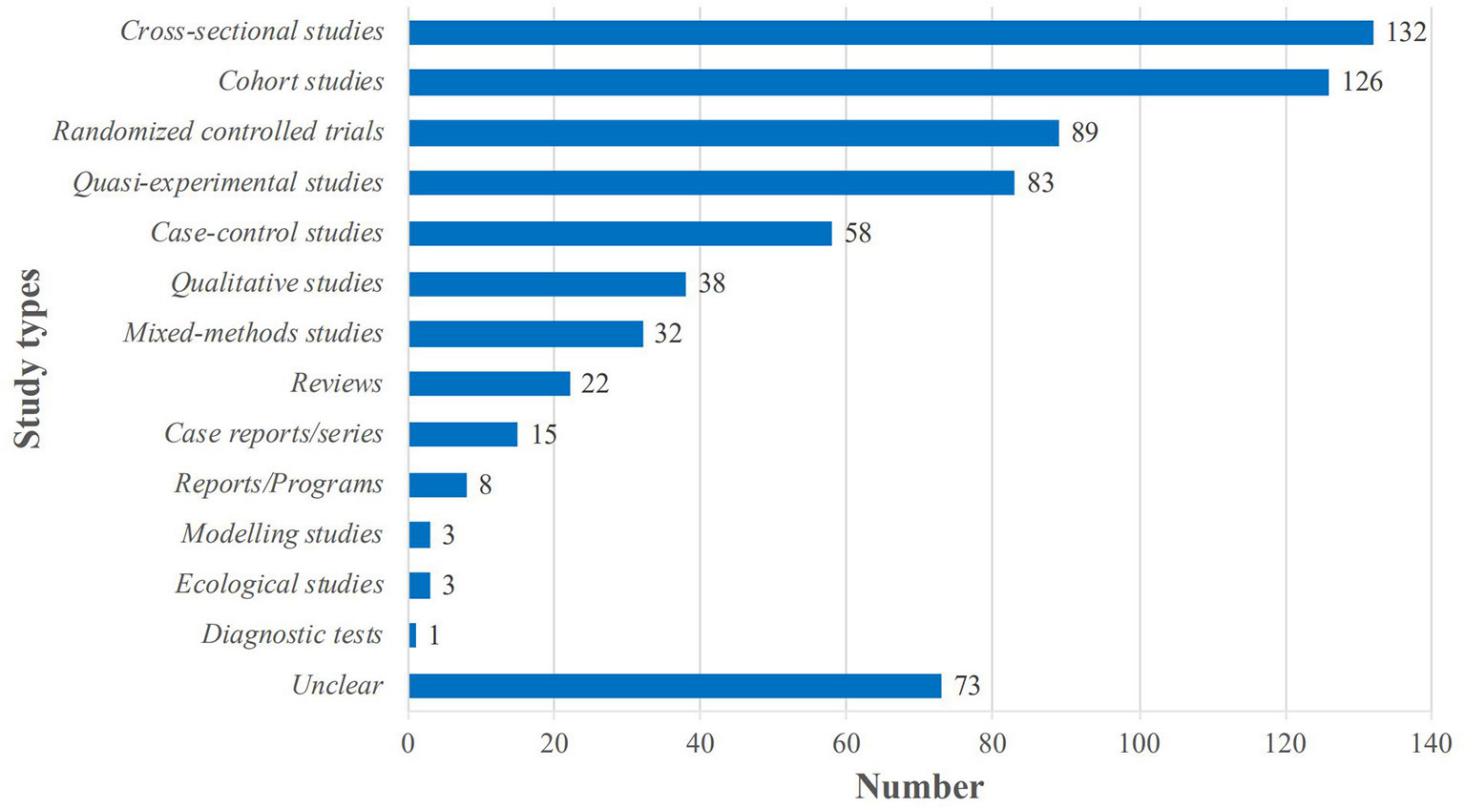
Distribution of the types of primary studies in the included systematic reviews (*n* = 301). Quasi-experimental studies including non-randomized trials and before and after studies; Case-control studies including nested case-control studies, case-cohort studies and case-crossover studies; “Unclear” means that it is not possible to identify the specific type of study, such as descriptions studies, observational studies, prospective studies, and retrospective studies and so on.

The most common types of primary studies included in the SRs were cross-sectional studies (*n* = 132, 43.8%); cohort studies (*n* = 126, 41.9%); RCTs (*n* = 89, 29.6%); quasi-experimental studies including non-randomized trials and before and after studies (*n* = 83, 27.6%); and case-control studies including nested case-control studies, case-cohort studies and case-crossover studies (*n* = 58, 19.3%); qualitative studies (*n* = 38, 12.6%) and mixed-methods studies (*n* = 32, 10.6%). In addition, 73 of the SRs (24.2%) included other types of studies such as descriptive studies, observational studies, prospective studies, and retrospective studies. Details are shown in Figure 2.

### 3.4. Methodological quality assessment tools

Table 3 shows the main methodological quality assessment tools (The frequency of use was ≥2) used for different study types. The most frequently used tools were NOS (used for 50.0% of cohort studies and 55.6% of case-control studies), ROB (50.7% of RCTs) and CASP (38.5% of qualitative studies). The median score for cohort studies assessed with NOS was 7.0 (possible maximum score 9), indicating low risk of bias. “Comparability” and “Adequacy of follow up of cohorts” were the items least frequently assessed or reported (Appendix 2). The median score reported with the NOS for case-control studies was 7.0 (possible maximum score 9), also indicating low risk of bias. “Selection of Controls” and “Non-Response rate” were the item least frequently assessed or reported (Appendix 3). The median score for RCTs assessed with ROB was 4.0 (possible maximum score 7), meaning therisk of bias was moderate. “Allocation concealment”, “Blinding of participants and personnel” and “Blinding of outcome assessment” were the least frequently assessed or reported items (Appendix 4). The median score for qualitative studies assessed with CASP was 9.0, out of a possible maximum score of 10. It was at low risk of bias. “Personal biases” and “Ethical considerations” were the items least frequently assessed or reported (Appendix 5). Five SRs used reporting checklists or an evidence grading system to evaluate the methodological quality.

**Table 3:** Methodological quality assessment tools for primary studies included in the systematic reviews by study type^*^.

**Methodological quality assessment tool**	**Number**	**%**
**Cross-sectional studies (*****N*** = **84)**		
NOS adaptation	14	16.7
NHLBI	9	10.7
JBI	7	8.3
AHRQ	4	4.8
ROBINS-I	4	4.8
Assessing risk of bias in prevalence studies	3	3.6
Assessing bias in studies of prognostic factors	3	3.6
EPHPP	3	3.6
AXIS	2	2.4
CASP	2	2.4
MMAT	2	2.4
STROBE	2	2.4
The Downs and Black checklist	2	2.4
**Cohort studies (*****N*** = **74)**		
NOS	37	50.0
NHLBI	5	6.7
EPHPP	5	6.7
ROBINS-I	3	4.0
JBI	3	4.0
Assessing bias in studies of prognostic factors	2	2.7
The Downs and Black checklist	2	2.7
**Randomized controlled trials (*****N*** = **67)**		
ROB	34	50.7
EPHPP	7	10.4
EPOC	3	4.5
JBI	3	4.5
ROB 2.0	3	4.5
MMAT	2	3.0
NHLBI	2	3.0
PEDro scale	2	3.0
**Quasi-experimental studies (*****N*** = **60)**		
ROB	10	16.7
EPHPP	9	15.0
JBI	6	10.0
The Downs and Black checklist	6	10.0
ROBINS-I	5	8.3
NHLBI	3	5.0
NOS	3	5.0
EPOC	2	3.3
MMAT	2	3.3
**Case-control studies (*****N*** = **45)**		
NOS	25	55.6
ROBINS-I	3	6.7
NHLBI	3	6.7
EPHPP	2	4.4
QUADAS-2	2	4.4
**Qualitative studies (*****N*** = **26)**		
CASP	10	38.5
MMAT	3	11.5
JBI	2	7.7
**Mixed-methods studies (*****N*** = **19)**		
MMAT	4	21.0
CASP	2	10.5
NHLBI	2	10.5

^*^We only present the most common quality evaluation tools (frequency of use ≥2). We only extracted data that were reported in the SRs.

AHRQ, Agency for Healthcare Research and Quality; AXIS, Appraisal Tool for Cross-Sectional Studies; CASP, Critical Appraisal Skills Programme; EPHPP, Effective Public Health Practice Project; EPOC, Effective Practice and Organization of Care Group; JBI, Joanna Briggs Institute; MMAT, Mixed Methods Appraisal Tool; NHLBI, National Heart, Lung and Blood Institute; NOS, Newcastle-Ottawa scale; PEDro scale, Physical Therapy Evidence Database scale; QUADAS-2, Quality Assessment of Diagnostic Accuracy Studies; ROB, The Cochrane Collaboration's tool for assessing risk of bias in randomized trials; ROBINS-I, Risk of bias in non-randomized studies of interventions; STROBE, Strengthening the Reporting of Observational Studies in Epidemiology.

### 3.5. Grading of certainty of evidence

Only 6.6% of the SRs (*n* = 20) assessed the certainty of the body of evidence for selected outcomes. The vast majority of these (*n* = 19, 95.0%) used the Grading of Recommendations, Assessment, Development, and Evaluation (GRADE) approach, while one study used the Oxford Center for Evidence-based Medicine (OCEBM) system (Table 4). The SRs that used GRADE evaluated a total of 111 outcomes: the certainty of the evidence was assessed as high for 11 (9.9%), moderate for 27 (24.3%), low for 28 (25.3%), and very low for 45 (40.5%) (Table 4). It is important to note that when reporting results for the GRADE assessments, these assessments are made at the level of individual outcomes, and some included reviews maybe contribute more than one assessment to this data. Ten (11.6%) of the 86 SRs of interventionsgraded the certainty of evidence including a total of 78 outcomes. The certainty of the evidence was high in11 (14.1%), moderate for 23 (29.5%), low for 16 (20.5%) and very low for 28 (35.9%).

**Table 4:** Approaches used to assess the certainty of the body of evidence in the systematic reviews.

**Title of the systematic review**	**Approach for assessing the body of evidence**	**GRADE assessment (number of outcomes)** ^**a**^			
		**High**	**Moderate**	**Low**	**Very low**
Vaccination among HIV-infected, HIV-exposed uninfected and HIV-uninfected children: a systematic review and meta-analysis of evidence related to vaccine efficacy and effectiveness	GRADE	4	1	1	3
Prevalence of strongyloidiasis and schistosomiasis among migrants: a systematic review and meta-analysis	GRADE	0	1	4	0
Effects of Housing First approaches on health and wellbeing of adults who are homeless or at risk of homelessness: systematic review and meta-analysis of randomized controlled trials	GRADE	0	4	4	0
Tele-ultrasound in resource-limited settings: a systematic review	GRADE	0	0	1	0
Relationship between caffeine intake and infertility: a systematic review of controlled clinical studies	GRADE	0	0	1	0
Zumba ^®^, fat mass and maximum oxygen consumption: a systematic review and meta-analysis	GRADE	0	0	1	0
Relationship between exposure to mixtures of persistent, bioaccumulative, and toxic chemicals and cancer risk: a systematic review	GRADE	0	1	0	0
Current strategies and successes in engaging women in vector control: a systematic review	GRADE	6	13	3	1
Do technical aids for patient handling prevent musculoskeletal complaints in health care workers? A systematic review of intervention studies	GRADE	0	0	1	4
Gender-related differences in care-seeking behavior for newborns: a systematic review of the evidence in South Asia	GRADE	0	0	1	1
The impact of financial incentives on physical activity: a systematic review and meta-analysis	GRADE	1	3	3	2
Physical activity interventions in faith-based organizations: a systematic review	GRADE	0	0	0	1
The effectiveness and cost-effectiveness of screening for HIV in migrants in the EUEEA: a systematic review	GRADE	0	1	1	0
The most effective amount of forward movement for oral appliances for obstructive sleep apnea: a systematic review	GRADE	0	0	3	5
Conference equity in global health: a systematic review of factors impacting LMIC representation at global health conferences	GRADE	0	0	0	15
Socioeconomic status throughout life and body mass index: a systematic review and meta-analysis	GRADE	0	0	0	1
Does short message service improve focused antenatal care visit and skilled birth attendance? A systematic review and meta-analysis of randomized clinical trials	GRADE	0	2	0	0
Corticosteroids on the management of coronavirus disease 2019 (COVID-19): a systemic review and meta-analysis	GRADE	0	0	0	12
Utilization of public health care by people with private health insurance: a systematic review and meta-analysis	GRADE	0	1	4	0
Burnout in palliative care nurses, prevalence and risk factors: a systematic review with meta-analysis	OCEBM	NA	NA	NA	NA
Total	-	11	27	28	45

^a^For systematic reviews that used GRADE to assess the quality of the body of evidence for critical or important outcomes, the number of outcomes assessed as high, moderate, low or very low certainty of evidence is presented.

Only 20 out of the 301 included systematic reviews assessed the certainty of the body of evidence. We would like to point out that when reporting results for the GRADE assessments, these assessments are made at the level of individual outcomes, and some included reviews may thus contribute more than one assessment.

GRADE, Grading of Recommendations, Assessment, Development, and Evaluation approach; OCEBM, Oxford Center for Evidence-based Medicine; NA, Not applicable.

## 4. Discussion

### 4.1. Summary of main results

The main sources of evidence in a random sample of SRs in public health were electronic bibliographic databases. Other sources of evidence were used by < 20% of the SRs. Cross-sectional studies represented more than a third of the primary studies included in the SRs, followed by cohort studies and RCTs. While more than 70% of the SRs evaluated the quality of the included studies using a broad range of quality assessment tools, only 6% assessed the certainty of the body of evidence. Of SRs on interventions, 11% graded the certainty of evidence. More than three quarters of the evidence assessed by GRADE was found to be of low or very low certainty.

### 4.2. Sources of evidence

A well-conducted systematic analyzes all available evidence to answer a carefully formulated question. It employs an objective search of the literature, applies predetermined inclusion and exclusion criteria, and critically appraises what is found to be relevant. However, systematic reviews may differ in quality, and yield different answers to the same question. As a result, users of systematic reviews should be critical and look carefully at the methodological quality of the available reviews. AMSTAR (updated to AMSTAR 2 in 2017) has been proven to be a reliable and valid measurement tool for assessing the methodological quality of systematic reviews (20–22). Item 3 in AMSTAR and item 4 in AMSTAR 2 state that authors need to use a comprehensive literature search strategy specifically requiring that at least two bibliographic databases should be searched. Searches should be supplemented by checking published reviews, specialized registers, or contacting experts in the particular field of study, and by reviewing the reference lists of the identified studies. Sometimes it is necessary to search websites (e.g., government agencies, non-governmental organizations or health technology agencies), trial registries, conference abstracts, dissertations, and unpublished reports on personal websites (e.g., universities, ResearchGate). In addition, PRISMA-S, an extension to the PRISMA Statement for Reporting Literature Searches in Systematic Reviews, covers multiple aspects of the search process for systematic reviews and presents a 16-item checklist similar to the AMSTAR (23).

Our study found that the median number of databases searched for each SR was four. To gain a comprehensive collection of research evidence on public health topics, it is necessary to search extensively also beyond Medline, including topic-specific databases as appropriate (24, 25). Although many scholars consider Medline as the most essential source of medical literature (24, 26). It does not exhaustively cover all the evidence related to public health, particularly publications with a regional or local focus. Therefore, researchers are encouraged to consider topic-specific databases (27). Other important sources for evidence in public health include reports from research organizations, governments and public health agencies, which are often not published in peer-reviewed journals, and can only be found on the organization's website (28). However, our findings show that most of the SRs did not supplement the evidence through other sources. Researchers are therefore encouraged to consider additional sources to retrieve evidence, such as registration platforms, conference proceedings, or contacting authors of key publications.

### 4.3. Types of evidence and methodological quality assessment tools

SRs of public health literature encompass evidence from a wide range of study designs, which is consistent with the complexity of implementing and evaluating public health interventions. Evidence on public health topics is therefore often derived from cross-sectional studies and quasi-experimental studies (6, 7, 29).

Our finding that approximately 70% of SRs evaluated the quality of the included primary studies is lower than what was found by a previous survey of medical journals, reporting that 90% of SRs evaluated the quality of included studies (30). AMSTAR 2 contains an item asking whether the review authors made an adequate assessment of study level efforts to avoid, control, or adjust for baseline confounding, selection bias, bias in measurement of exposures and outcomes, and selective reporting of analyses or outcomes. Using a satisfactory technique for assessing the risk of bias in individual studies that were included in the review is critical (20).

Given the large number of study designs used in public health research, many quality assessment tools are available and there is little consensus on the optimal tool(s) for each specific study designs. For example, for cross-sectional studies and other observational studies, nearly one hundred tools are available (31–35). We also found that researchers did not always correctly distinguish the concepts of methodological quality from reporting quality. For instance, some researchers used reporting checklists (e.g., STROBE, Strengthening the Reporting of Observational Studies in Epidemiology) to evaluate the methodological quality of cohort studies (36) and cross-sectional studies (37, 38). Zeng et al. also reported similar problems (31, 32). Therefore, our findings suggest that SR developers may need additional training and guidance on the selection of optimal tools for assessing the quality of primary studies, particularly non-randomized studies.

### 4.4. Grading of certainty of evidence

The certainty of the body of evidence refers to the degree of certainty about the veracity of the observations for a specific outcome. Assessing the certainty of a body of evidence form a SR can facilitate an accurate understanding and appropriate application of the evidence by end-users (39). Thus, our finding that only ~6% of SRs assessed the certainty of the body of evidence is concerning. The main reason may be that public health interventions are often complex, making it difficult to perform such assessments (40, 41). Researchers have found several problems in application of GRADE, currently the most widely used approach for assessing the quality or certainty of the body of evidence in public health: confusion about the perspectives of different stakeholders, selection of outcomes and identification of different sources of evidence (42), and the non-applicability of the specific terminology of GRADE in the field of public health (43). In response to these issues, the GRADE Working Group has established the GRADE Public Health Group (42). In addition, we noted that some SR authors misunderstood the principles of GRADE and applied it at the study level rather than the outcome level (44). Thus, researchers performing SRs may need additional experience and training on the use of GRADE to facilitate its correct and rational use.

Most of the evidence in the SRs that applied GRADE was found to be of low or very low certainty, which may be related to a high risk of bias in the primary studies, heterogeneity of results across the body of evidence, or directness, i.e., that the evidence does not apply directly to the key question of the SR.

### 4.5. Strength and limitations

To our knowledge, this is the first comprehensive examination of the sources, types and quality of evidence used in SRs of the public health literature. The main strength of this study is the rigorous application of systematic methods in the literature search, screening, and data extraction. Moreover, our study sample (*n* = 301) is large, it was sampled randomly from all eligible SRs and is up to date, containing SRs published in the last 3 years.

Our study has also some limitations. Our results reflect what was reported in the articles, and it is possible that some SRs were conducted more rigorously than how they were reported, or *vice versa*. Additional information could have been gleaned by contacting the authors of the SRs or by examining the primary studies themselves. Our study only analyzed literature published in English, the findings of this study may not apply to SRs published in other languages. Finally, we used the study design designation made by the SR authors, given the variability of terminology, and it is possible that the types of some studies were misclassified.

## 5. Conclusion

SRs should always assess quality both at the individual study level, and the level of the entire body of evidence. Investigators in public health need to focus on the robustness of the study design, minimize the risk of bias to the largest possible extent, and comprehensively report the methods and findings of their studies.

## Data availability statement

The original contributions presented in the study are included in the article/Supplementary material, further inquiries can be directed to the corresponding authors.

## Author contributions

YX, QG, YC, and RS developed the concept of the study. QG, YX, MR, YL, YS, SW, HLa, JZ, HLi, and PW were responsible for data curation. QG and YX analyzed the data and wrote the original draft of the manuscript. YX, JW, QS, QW, YC, and RS reviewed and edited the manuscript. All authors read and agreed the final submitted manuscript.
