# Supplementary material for: Characteristics of the sources, evaluation, and grading of the certainty of evidence in systematic reviews in public health: A methodological study
Source: Front Public Health. 2023 Mar 30;11:998588. doi: 10.3389/fpubh.2023.998588 (PMC10097925; doi:10.3389/fpubh.2023.998588)
Supplement: Supplementary file 1 [file Table_1.DOCX]

**Appendix 2** Item scores for the Newcastle-Ottawa Scale tool used for cohort studies

| Title of the systematic review | Representativeness of the exposed cohort | Selection of the non exposed cohort | Ascertainment of exposure | Demonstration that outcome of interest was not present at start of study | Comparability* | Assessment of outcome | Was follow-up long enough for outcomes to occur | Adequacy of follow up of cohorts | Total score |
| --- | --- | --- | --- | --- | --- | --- | --- | --- | --- |
| Relationship between caffeine intake and infertility: a systematic review of controlled clinical studies | 1 | 1 | 1 | 1 | 2 | 1 | 1 | 1 | 9 |
| Association between Fruit and Vegetable Consumption and Depression Symptoms in Young People and Adults Aged 15-45: A Systematic Review of Cohort Studies | 1 | 1 | 0 | 1 | 2 | 0 | 1 | 1 | 7 |
|  | 1 | 1 | 0 | 1 | 2 | 0 | 1 | 1 | 7 |
|  | 1 | 1 | 0 | 0 | 2 | 0 | 1 | 1 | 6 |
|  | 1 | 1 | 0 | 1 | 2 | 0 | 1 | 0 | 6 |
|  | 0 | 1 | 0 | 1 | 2 | 0 | 1 | 1 | 6 |
|  | 1 | 1 | 0 | 1 | 2 | 0 | 1 | 0 | 6 |
|  | 0 | 1 | 0 | 1 | 2 | 0 | 1 | 1 | 6 |
|  | 0 | 1 | 0 | 1 | 2 | 0 | 1 | 0 | 5 |
|  | 1 | 1 | 1 | 1 | 2 | 0 | 1 | 1 | 8 |
|  | 0 | 1 | 0 | 1 | 2 | 0 | 1 | 1 | 6 |
|  | 1 | 0 | 0 | 0 | 2 | 0 | 1 | 0 | 4 |
|  | 0 | 1 | 0 | 1 | 2 | 0 | 1 | 1 | 6 |
| Maternal lifestyle characteristics and Wilms tumor risk in the offspring: A systematic review and meta-analysis | 1 | 1 | 0 | 1 | 0 | 1 | 1 | 1 | 6 |
|  | 1 | 1 | 0 | 1 | 1 | 1 | 1 | 1 | 7 |
|  | 1 | 1 | 1 | 1 | 2 | 1 | 1 | 1 | 9 |
|  | 1 | 1 | 0 | 0 | 2 | 0 | 1 | 1 | 6 |
|  | 1 | 1 | 1 | 1 | 2 | 1 | 1 | 1 | 9 |
| Relationship between exposure to mixtures of persistent, bioaccumulative, and toxic chemicals and cancer risk: A systematic review | 1 | 1 | 1 | 1 | 2 | 1 | 0 | 1 | 8 |
| Association of oral health literacy with oral health behaviors, perception, knowledge, and dental treatment related outcomes: a systematic review and meta-analysis | 0 | 1 | 1 | 1 | 2 | 1 | 1 | 1 | 8 |
|  | 0 | 1 | 1 | 1 | 2 | 1 | 1 | 1 | 8 |
| Dietary Heterocyclic Amine Intake and Colorectal Adenoma Risk: A Systematic Review and Meta-analysis | 1 | 1 | 1 | 1 | 2 | 1 | 1 | 0 | 8 |
|  | 1 | 1 | 1 | 1 | 2 | 1 | 1 | 0 | 8 |
|  | 1 | 1 | 1 | 1 | 2 | 1 | 1 | 0 | 8 |
| Relationship between particulate matter exposure and female breast cancer incidence and mortality: a systematic review and meta-analysis | 1 | 1 | 1 | 1 | 2 | 1 | 0 | 0 | 7 |
|  | 1 | 1 | 1 | 1 | 2 | 1 | 1 | 1 | 9 |
|  | 0 | 1 | 1 | 1 | 2 | 1 | 1 | 1 | 8 |
|  | 0 | 1 | 1 | 1 | 2 | 1 | 1 | 1 | 8 |
|  | 0 | 1 | 1 | 1 | 2 | 1 | 1 | 1 | 8 |
|  | 1 | 1 | 1 | 1 | 2 | 1 | 1 | 1 | 9 |
|  | 0 | 1 | 1 | 1 | 2 | 1 | 1 | 1 | 8 |
|  | 1 | 1 | 1 | 1 | 2 | 1 | 0 | 1 | 8 |
|  | 0 | 1 | 1 | 1 | 2 | 1 | 1 | 1 | 8 |
|  | 1 | 1 | 1 | 1 | 2 | 1 | 1 | 1 | 9 |
|  | 1 | 1 | 1 | 1 | 2 | 1 | 1 | 1 | 9 |
|  | 1 | 0 | 1 | 1 | 1 | 1 | 0 | 1 | 6 |
|  | 1 | 1 | 1 | 1 | 1 | 1 | 0 | 1 | 7 |
| Occupational exposure to formaldehyde and risk of lung cancer: A systematic review and meta‐analysis | 1 | 0 | 0 | 0 | 1 | 1 | 1 | 0 | 4 |
|  | 1 | 1 | 0 | 0 | 1 | 1 | 1 | 0 | 5 |
|  | 1 | 1 | 1 | 0 | 1 | 1 | 1 | 0 | 6 |
|  | 1 | 1 | 0 | 0 | 1 | 1 | 1 | 0 | 5 |
|  | 1 | 1 | 0 | 0 | 1 | 1 | 1 | 1 | 6 |
|  | 1 | 1 | 1 | 0 | 1 | 1 | 1 | 0 | 6 |
|  | 1 | 1 | 0 | 0 | 2 | 1 | 0 | 0 | 5 |
|  | 1 | 1 | 1 | 0 | 1 | 1 | 1 | 0 | 6 |
|  | 1 | 1 | 1 | 0 | 1 | 1 | 1 | 1 | 7 |
|  | 1 | 1 | 1 | 0 | 2 | 1 | 1 | 0 | 7 |
|  | 1 | 1 | 1 | 0 | 1 | 1 | 1 | 0 | 6 |
|  | 1 | 1 | 1 | 0 | 1 | 1 | 1 | 0 | 6 |
|  | 1 | 1 | 1 | 0 | 1 | 1 | 1 | 0 | 6 |
|  | 1 | 1 | 0 | 0 | 1 | 1 | 1 | 0 | 5 |
| Association between anaemia and adult depression: a systematic review and meta-analysis of observational studies | 1 | 1 | 1 | 1 | 2 | 1 | 0 | 1 | 8 |
|  | 1 | 1 | 0 | 0 | 1 | 1 | 0 | 0 | 4 |
|  | 0 | 0 | 1 | 1 | 2 | 1 | 0 | 1 | 6 |
|  | 1 | 1 | 1 | 1 | 2 | 1 | 1 | 0 | 8 |
|  | 1 | 1 | 1 | 1 | 1 | 1 | 0 | 0 | 6 |
| Association between prenatal exposure to ambient particulate matter and risk of hypospadias in offspring: A systematic review and meta-analysis | 1 | 1 | 1 | 1 | 2 | 1 | 1 | 1 | 9 |
|  | 1 | 1 | 1 | 1 | 1 | 1 | 1 | 1 | 8 |
| Association of malnutrition with periprosthetic joint and surgical site infections after total joint arthroplasty: a systematic review and meta-analysis | 1 | 1 | 1 | 1 | 1 | 1 | 1 | 0 | 7 |
|  | 1 | 1 | 1 | 1 | 1 | 1 | 1 | 0 | 7 |
|  | 0 | 1 | 1 | 1 | 0 | 0 | 1 | 0 | 4 |
|  | 1 | 1 | 1 | 1 | 1 | 1 | 1 | 0 | 7 |
|  | 1 | 1 | 1 | 1 | 2 | 1 | 1 | 0 | 8 |
|  | 0 | 1 | 1 | 1 | 2 | 1 | 1 | 0 | 7 |
|  | 0 | 1 | 1 | 1 | 2 | 1 | 1 | 0 | 7 |
|  | 1 | 1 | 1 | 1 | 0 | 0 | 1 | 0 | 5 |
| HIV Nonoccupational Postexposure Prophylaxis Among Men Who Have Sex with Men: A Systematic Review and Meta-Analysis of Global Data | 0 | 0 | 1 | 1 | 0 | 1 | 1 | 1 | 5 |
|  | 1 | 1 | 1 | 1 | 1 | 1 | 1 | 1 | 8 |
|  | 1 | 0 | 1 | 1 | 0 | 1 | 1 | 1 | 6 |
|  | 1 | 0 | 1 | 1 | 0 | 1 | 1 | 1 | 6 |
|  | 1 | 0 | 1 | 1 | 0 | 1 | 1 | 1 | 6 |
|  | 1 | 1 | 1 | 1 | 2 | 1 | 1 | 1 | 9 |
|  | 1 | 0 | 1 | 1 | 0 | 1 | 1 | 0 | 5 |
|  | 1 | 1 | 1 | 1 | 2 | 1 | 1 | 0 | 8 |
|  | 0 | 1 | 1 | 1 | 0 | 1 | 1 | 0 | 5 |
|  | 1 | 1 | 1 | 1 | 1 | 1 | 1 | 0 | 7 |
|  | 0 | 1 | 1 | 1 | 2 | 1 | 1 | 1 | 8 |
|  | 1 | 0 | 1 | 1 | 0 | 1 | 1 | 0 | 5 |
|  | 1 | 0 | 1 | 1 | 0 | 0 | 1 | 1 | 5 |
|  | 0 | 0 | 1 | 1 | 0 | 1 | 1 | 1 | 5 |
| Exposure to air pollution and cognitive impairment risk: a meta-analysis of longitudinal cohort studies with dose-response analysis | 1 | 0 | 1 | 1 | 2 | 1 | 1 | 1 | 8 |
|  | 1 | 0 | 1 | 1 | 2 | 1 | 1 | 1 | 8 |
|  | 1 | 0 | 1 | 1 | 2 | 1 | 1 | 1 | 8 |
|  | 1 | 0 | 1 | 1 | 2 | 1 | 1 | 1 | 8 |
|  | 1 | 0 | 1 | 1 | 2 | 1 | 1 | 1 | 8 |
|  | 1 | 0 | 1 | 0 | 2 | 1 | 1 | 1 | 7 |
|  | 1 | 0 | 1 | 1 | 2 | 1 | 1 | 1 | 8 |
|  | 1 | 0 | 1 | 1 | 2 | 1 | 1 | 1 | 8 |
|  | 1 | 0 | 1 | 1 | 2 | 1 | 1 | 1 | 8 |
|  | 1 | 0 | 1 | 1 | 2 | 1 | 1 | 1 | 8 |
| Association between exposure to air pollutants and attention-deficit hyperactivity disorder (ADHD) in children: a systematic review and meta- analysis | 1 | 1 | 1 | 1 | 1 | 1 | 1 | 0 | 7 |
|  | 1 | 1 | 1 | 1 | 1 | 0 | 0 | 0 | 5 |
|  | 1 | 1 | 1 | 1 | 1 | 1 | 1 | 0 | 7 |
|  | 1 | 1 | 1 | 1 | 1 | 1 | 1 | 1 | 8 |
|  | 1 | 1 | 1 | 1 | 1 | 1 | 1 | 0 | 7 |
|  | 1 | 1 | 1 | 1 | 1 | 1 | 1 | 0 | 7 |
|  | 1 | 1 | 1 | 1 | 1 | 1 | 1 | 0 | 7 |
| **Total score** | **77** | **75** | **76** | **78** | **140** | **80** | **86** | **55** | **M (7)** |

*: “Comparability” means study controls for the most important factor and a second important factor. A maximum of value “2” can be given for “Comparability”.

Legend: Each row represents a primary study that was included in the systematic review. Each cell is either “1” when the answer to the corresponding question was “yes” or “partial yes” or “0” if the answer was “no” or “cannot tell”.

It is worth noting that items were analyzed based on the assessment of the authors of the included reviews. Based on the detailed item score of the used assessment tool of the included reviews, we re-analyzed the methodological quality of primary studies of the included reviews.
