# Supplementary material for: Characteristics of the sources, evaluation, and grading of the certainty of evidence in systematic reviews in public health: A methodological study
Source: Front Public Health. 2023 Mar 30;11:998588. doi: 10.3389/fpubh.2023.998588 (PMC10097925; doi:10.3389/fpubh.2023.998588)
Supplement: Supplementary file 2 [file Table_2.DOCX]

**Appendix 3** Item scores for the Newcastle-Ottawa Scale tool used for case-control studies

| Title of the systematic review | Adequate case definition | Representativeness of the cases | Selection of Controls | Definition of Controls | Comparability* | Ascertainment of exposure | Same method of ascertainment for cases and controls | Non-Response rate | Total score |
| --- | --- | --- | --- | --- | --- | --- | --- | --- | --- |
| Relationship between caffeine intake and infertility: a systematic review of controlled clinical studies | 1 | 1 | 1 | 1 | 0 | 1 | 1 | 0 | 6 |
|  | 1 | 1 | 0 | 1 | 1 | 0 | 1 | 1 | 6 |
|  | 1 | 1 | 0 | 1 | 2 | 0 | 1 | 1 | 7 |
| Maternal lifestyle characteristics and Wilms tumor risk in the offspring: A systematic review and meta-analysis | 1 | 1 | 1 | 1 | 2 | 0 | 1 | 1 | 8 |
|  | 1 | 1 | 1 | 1 | 2 | 1 | 1 | 1 | 9 |
|  | 1 | 1 | 0 | 1 | 2 | 1 | 1 | 1 | 8 |
|  | 1 | 1 | 1 | 1 | 2 | 0 | 1 | 0 | 7 |
|  | 1 | 1 | 1 | 1 | 2 | 1 | 1 | 0 | 8 |
|  | 1 | 1 | 1 | 1 | 2 | 1 | 1 | 1 | 9 |
|  | 1 | 1 | 1 | 1 | 2 | 1 | 1 | 0 | 8 |
|  | 1 | 1 | 1 | 1 | 2 | 1 | 1 | 0 | 8 |
|  | 1 | 1 | 0 | 1 | 2 | 1 | 1 | 1 | 8 |
|  | 1 | 1 | 0 | 1 | 2 | 1 | 1 | 1 | 8 |
|  | 1 | 1 | 0 | 1 | 1 | 0 | 1 | 1 | 6 |
|  | 1 | 1 | 1 | 1 | 2 | 1 | 1 | 0 | 8 |
| Relationship between exposure to mixtures of persistent, bioaccumulative, and toxic chemicals and cancer risk: A systematic review | 1 | 1 | 0 | 1 | 2 | 1 | 1 | 1 | 8 |
|  | 1 | 1 | 1 | 0 | 2 | 1 | 1 | 0 | 7 |
|  | 1 | 1 | 0 | 0 | 2 | 1 | 1 | 0 | 6 |
|  | 1 | 1 | 1 | 1 | 2 | 1 | 1 | 1 | 9 |
|  | 1 | 1 | 1 | 1 | 2 | 1 | 1 | 0 | 8 |
|  | 1 | 1 | 0 | 1 | 2 | 1 | 1 | 0 | 7 |
|  | 0 | 0 | 0 | 0 | 1 | 1 | 1 | 0 | 3 |
|  | 1 | 1 | 1 | 1 | 1 | 1 | 1 | 0 | 7 |
|  | 1 | 1 | 0 | 0 | 2 | 1 | 1 | 0 | 6 |
|  | 1 | 1 | 1 | 1 | 2 | 1 | 1 | 1 | 9 |
|  | 1 | 1 | 0 | 1 | 1 | 1 | 1 | 0 | 6 |
|  | 1 | 1 | 0 | 1 | 2 | 1 | 1 | 1 | 8 |
|  | 0 | 1 | 0 | 1 | 2 | 1 | 1 | 0 | 6 |
|  | 1 | 1 | 1 | 1 | 1 | 1 | 1 | 0 | 7 |
|  | 1 | 1 | 0 | 1 | 2 | 1 | 1 | 1 | 8 |
|  | 1 | 1 | 1 | 1 | 2 | 1 | 1 | 1 | 9 |
|  | 1 | 1 | 0 | 1 | 2 | 1 | 1 | 0 | 7 |
|  | 1 | 1 | 1 | 1 | 2 | 1 | 1 | 0 | 8 |
|  | 1 | 1 | 1 | 1 | 2 | 1 | 1 | 1 | 9 |
|  | 1 | 1 | 0 | 1 | 2 | 1 | 1 | 0 | 7 |
|  | 1 | 1 | 1 | 1 | 2 | 1 | 1 | 1 | 9 |
| Association of oral health literacy with oral health behaviors, perception, knowledge, and dental treatment related outcomes: a systematic review and meta-analysis | 1 | 1 | 1 | 1 | 2 | 1 | 1 | 0 | 8 |
| Dietary Heterocyclic Amine Intake and Colorectal Adenoma Risk: A Systematic Review and Meta-analysis | 1 | 1 | 0 | 1 | 2 | 1 | 0 | 0 | 6 |
|  | 1 | 1 | 1 | 1 | 2 | 1 | 1 | 1 | 9 |
|  | 1 | 1 | 0 | 1 | 2 | 1 | 0 | 0 | 6 |
|  | 1 | 1 | 1 | 1 | 2 | 1 | 1 | 1 | 9 |
|  | 1 | 1 | 1 | 1 | 2 | 1 | 1 | 1 | 9 |
|  | 1 | 1 | 1 | 1 | 2 | 1 | 1 | 1 | 9 |
|  | 1 | 1 | 1 | 1 | 2 | 1 | 1 | 1 | 9 |
|  | 1 | 1 | 1 | 1 | 2 | 1 | 1 | 1 | 9 |
|  | 1 | 1 | 1 | 1 | 2 | 1 | 1 | 1 | 9 |
| Relationship between particulate matter exposure and female breast cancer incidence and mortality: a systematic review and meta‐analysis | 1 | 1 | 1 | 1 | 2 | 1 | 1 | 1 | 9 |
| Occupational exposure to formaldehyde and risk of lung cancer: A systematic review and meta‐analysis | 0 | 1 | 1 | 0 | 1 | 0 | 1 | 0 | 4 |
|  | 0 | 1 | 1 | 0 | 1 | 1 | 1 | 1 | 6 |
|  | 1 | 1 | 1 | 1 | 2 | 1 | 1 | 0 | 8 |
|  | 0 | 1 | 1 | 1 | 2 | 1 | 1 | 0 | 7 |
|  | 0 | 1 | 1 | 1 | 2 | 0 | 1 | 0 | 6 |
|  | 0 | 1 | 1 | 1 | 2 | 1 | 1 | 0 | 7 |
|  | 0 | 1 | 1 | 0 | 2 | 1 | 1 | 0 | 6 |
|  | 1 | 1 | 1 | 0 | 2 | 1 | 0 | 0 | 6 |
|  | 0 | 1 | 1 | 0 | 2 | 1 | 1 | 0 | 6 |
|  | 0 | 1 | 1 | 1 | 2 | 1 | 1 | 0 | 7 |
|  | 1 | 0 | 0 | 1 | 2 | 0 | 1 | 1 | 6 |
|  | 1 | 1 | 1 | 1 | 2 | 1 | 1 | 0 | 8 |
|  | 1 | 1 | 1 | 0 | 2 | 1 | 1 | 0 | 7 |
| Association between anaemia and adult depression: a systematic review and meta-analysis of observational studies | 1 | 0 | 1 | 0 | 2 | 1 | 1 | 0 | 6 |
|  | 1 | 1 | 1 | 0 | 2 | 1 | 1 | 0 | 7 |
|  | 1 | 1 | 1 | 0 | 2 | 1 | 1 | 0 | 7 |
|  | 1 | 1 | 1 | 0 | 2 | 1 | 1 | 0 | 7 |
|  | 1 | 1 | 1 | 0 | 2 | 1 | 1 | 0 | 7 |
|  | 1 | 1 | 1 | 0 | 2 | 1 | 1 | 0 | 7 |
|  | 1 | 1 | 1 | 0 | 1 | 0 | 1 | 0 | 5 |
|  | 1 | 1 | 1 | 0 | 1 | 0 | 1 | 0 | 5 |
|  | 1 | 1 | 1 | 0 | 2 | 1 | 1 | 0 | 7 |
| Association between prenatal exposure to ambient particulate matter and risk of hypospadias in offspring: A systematic review and meta-analysis | 1 | 1 | 0 | 1 | 1 | 1 | 1 | 1 | 7 |
|  | 1 | 1 | 1 | 1 | 2 | 1 | 1 | 1 | 9 |
|  | 1 | 1 | 0 | 1 | 2 | 1 | 1 | 1 | 8 |
|  | 1 | 1 | 1 | 1 | 1 | 1 | 1 | 1 | 8 |
|  | 1 | 1 | 0 | 1 | 2 | 1 | 1 | 1 | 8 |
|  | 1 | 1 | 1 | 1 | 2 | 1 | 1 | 1 | 9 |
|  | 1 | 1 | 1 | 1 | 2 | 1 | 1 | 1 | 9 |
| **Total score** | **66** | **73** | **53** | **57** | **138** | **66** | **73** | **34** | **M (7)** |

*: “Comparability” means study controls for the most important factor and a second important factor. A maximum of value “2” can be given for “Comparability”.

Legend: Each row represents a primary study that was included in the systematic review. Each cell is either “1” when the answer to the corresponding question was “yes” or “partial yes” or “0” if the answer was “no” or “cannot tell”.

It is worth noting that items were analyzed based on the assessment of the authors of the included reviews. Based on the detailed item score of the used assessment tool of the included reviews, we re-analyzed the methodological quality of primary studies of the included reviews.
