# Supplementary material for: Characteristics of the sources, evaluation, and grading of the certainty of evidence in systematic reviews in public health: A methodological study
Source: Front Public Health. 2023 Mar 30;11:998588. doi: 10.3389/fpubh.2023.998588 (PMC10097925; doi:10.3389/fpubh.2023.998588)
Supplement: Supplementary file 3 [file Table_3.DOCX]

**Appendix 4** Item scores for the Cochrane Collaboration’s Risk of Bias tool used for randomized controlled trials

| Title of the systematic review | Random sequence generation | Allocation concealment | Blinding of participants and personnel | Blinding of outcome assessment | Incomplete outcome data | Selective reporting | Other bias | Total score |
| --- | --- | --- | --- | --- | --- | --- | --- | --- |
| Prevention of dental caries in Indigenous children from World Health Organization-listed high-income countries: A systematic review | 1 | 0 | 0 | 1 | 0 | 0 | 0 | 2 |
|  | 1 | 0 | 0 | 1 | 1 | 0 | 0 | 3 |
|  | 0 | 1 | 1 | 1 | 0 | 0 | 0 | 3 |
|  | 1 | 0 | 0 | 0 | 0 | 0 | 0 | 1 |
| The Effects of Chlorhexidine Dressing on Health Care-Associated Infection in Hospitalized Patients: A Meta-Analysis | 1 | 1 | 1 | 0 | 1 | 0 | 1 | 5 |
|  | 1 | 0 | 1 | 0 | 1 | 1 | 0 | 4 |
|  | 1 | 0 | 0 | 0 | 1 | 1 | 0 | 3 |
|  | 1 | 1 | 0 | 0 | 1 | 1 | 0 | 4 |
|  | 1 | 1 | 1 | 0 | 1 | 1 | 1 | 6 |
|  | 1 | 1 | 0 | 0 | 1 | 1 | 1 | 5 |
|  | 1 | 1 | 1 | 0 | 1 | 1 | 1 | 6 |
|  | 1 | 1 | 0 | 0 | 1 | 1 | 0 | 4 |
|  | 1 | 1 | 0 | 0 | 1 | 0 | 0 | 3 |
|  | 1 | 0 | 0 | 0 | 1 | 0 | 0 | 2 |
|  | 1 | 1 | 1 | 1 | 1 | 1 | 0 | 6 |
|  | 1 | 1 | 1 | 1 | 1 | 1 | 0 | 6 |
|  | 1 | 1 | 1 | 0 | 1 | 1 | 1 | 6 |
| Efficacy and cultural appropriateness of psychosocial interventions for paediatric burn patients and caregivers: a systematic review | 0 | 0 | 0 | 0 | 1 | 1 | 1 | 3 |
|  | 1 | 1 | 0 | 0 | 0 | 0 | 1 | 3 |
|  | 1 | 1 | 0 | 0 | 1 | 1 | 0 | 4 |
|  | 1 | 1 | 0 | 0 | 0 | 0 | 1 | 3 |
|  | 0 | 0 | 0 | 0 | 1 | 0 | 1 | 2 |
|  | 0 | 0 | 0 | 1 | 0 | 1 | 1 | 3 |
|  | 1 | 1 | 0 | 0 | 0 | 0 | 1 | 3 |
|  | 1 | 1 | 0 | 1 | 0 | 1 | 1 | 5 |
|  | 0 | 0 | 0 | 0 | 1 | 1 | 0 | 2 |
|  | 1 | 1 | 0 | 0 | 1 | 1 | 1 | 5 |
|  | 1 | 1 | 0 | 0 | 0 | 0 | 1 | 3 |
|  | 1 | 1 | 0 | 0 | 0 | 1 | 1 | 4 |
|  | 0 | 0 | 0 | 0 | 1 | 1 | 1 | 3 |
|  | 0 | 0 | 0 | 0 | 0 | 1 | 1 | 2 |
|  | 1 | 1 | 0 | 0 | 0 | 1 | 1 | 4 |
|  | 1 | 1 | 0 | 1 | 1 | 1 | 0 | 5 |
|  | 0 | 0 | 0 | 0 | 0 | 1 | 1 | 2 |
| Interventions to reduce unnecessary central venous catheter use to prevent central-line-associated bloodstream infections in adults: A systematic review | 1 | 0 | 0 | 1 | 1 | 1 | 1 | 5 |
| Reproductive factors and lung cancer risk: a comprehensive systematic review and meta-analysis | 1 | 1 | 1 | 1 | 1 | 1 | 0 | 6 |
|  | 1 | 1 | 1 | 1 | 0 | 1 | 0 | 5 |
|  | 1 | 1 | 1 | 0 | 1 | 1 | 0 | 5 |
|  | 1 | 0 | 1 | 1 | 1 | 1 | 0 | 5 |
| Corticosteroids on the Management of Coronavirus Disease 2019 (COVID-19): A Systemic Review and Meta-Analysis | 1 | 0 | 0 | 1 | 1 | 0 | 0 | 3 |
| Secnidazole for treatment of bacterial vaginosis: a systematic review | 1 | 0 | 1 | 0 | 1 | 0 | 0 | 3 |
|  | 1 | 0 | 1 | 1 | 1 | 1 | 0 | 5 |
|  | 1 | 1 | 1 | 1 | 1 | 1 | 0 | 6 |
|  | 0 | 0 | 0 | 0 | 0 | 0 | 0 | 0 |
|  | 0 | 0 | 1 | 1 | 1 | 1 | 0 | 4 |
|  | 1 | 0 | 1 | 0 | 0 | 0 | 0 | 2 |
| Health-Related Rehabilitation after the 2008 Great Wenchuan Earthquake in China: A Ten Year Retrospective Systematic Review | 1 | 1 | 0 | 0 | 0 | 1 | 0 | 3 |
|  | 1 | 0 | 0 | 0 | 0 | 1 | 0 | 2 |
|  | 0 | 0 | 0 | 0 | 0 | 1 | 0 | 1 |
|  | 1 | 0 | 0 | 1 | 1 | 1 | 0 | 4 |
| Effectiveness of communicative and educative strategies in chronic low back pain patients: a systematic review | 1 | 0 | 0 | 0 | 1 | 1 | 1 | 4 |
|  | 1 | 1 | 0 | 0 | 1 | 0 | 1 | 4 |
|  | 1 | 1 | 0 | 0 | 1 | 1 | 1 | 5 |
|  | 0 | 0 | 0 | 0 | 0 | 0 | 1 | 1 |
|  | 1 | 1 | 0 | 0 | 1 | 1 | 1 | 5 |
|  | 0 | 0 | 0 | 0 | 0 | 1 | 0 | 1 |
|  | 1 | 1 | 0 | 1 | 1 | 1 | 1 | 6 |
|  | 1 | 0 | 0 | 1 | 1 | 1 | 1 | 5 |
|  | 1 | 1 | 0 | 1 | 1 | 1 | 1 | 6 |
|  | 1 | 1 | 0 | 0 | 0 | 0 | 0 | 2 |
|  | 1 | 1 | 0 | 0 | 1 | 0 | 1 | 4 |
|  | 1 | 1 | 0 | 1 | 1 | 0 | 1 | 5 |
|  | 1 | 1 | 0 | 1 | 0 | 0 | 1 | 4 |
|  | 1 | 0 | 0 | 0 | 1 | 1 | 1 | 4 |
|  | 1 | 1 | 0 | 1 | 1 | 1 | 1 | 6 |
|  | 1 | 1 | 0 | 1 | 1 | 1 | 1 | 6 |
|  | 1 | 0 | 0 | 0 | 0 | 1 | 0 | 2 |
|  | 1 | 1 | 0 | 1 | 1 | 1 | 0 | 5 |
|  | 1 | 1 | 0 | 1 | 1 | 0 | 1 | 5 |
|  | 1 | 1 | 0 | 1 | 1 | 1 | 0 | 5 |
|  | 1 | 1 | 0 | 1 | 0 | 1 | 1 | 5 |
|  | 1 | 0 | 0 | 1 | 1 | 0 | 1 | 4 |
|  | 1 | 1 | 0 | 1 | 1 | 0 | 0 | 4 |
|  | 1 | 0 | 0 | 0 | 1 | 1 | 1 | 4 |
| The Methodological Quality and Effectiveness of Technology-Based Smoking Cessation Interventions for Disadvantaged Groups: A Systematic Review and Meta-analysis | 1 | 1 | 0 | 1 | 0 | 1 | 1 | 5 |
|  | 1 | 0 | 0 | 0 | 1 | 1 | 1 | 4 |
|  | 1 | 1 | 0 | 1 | 1 | 1 | 1 | 6 |
|  | 0 | 0 | 0 | 0 | 1 | 1 | 1 | 3 |
|  | 1 | 0 | 0 | 1 | 1 | 1 | 1 | 5 |
|  | 0 | 1 | 0 | 1 | 1 | 1 | 1 | 5 |
|  | 1 | 1 | 0 | 0 | 1 | 0 | 0 | 3 |
|  | 1 | 0 | 0 | 0 | 0 | 0 | 0 | 1 |
|  | 1 | 0 | 0 | 1 | 0 | 1 | 1 | 4 |
|  | 1 | 1 | 0 | 0 | 1 | 0 | 1 | 4 |
|  | 1 | 1 | 0 | 0 | 1 | 0 | 1 | 4 |
|  | 1 | 0 | 0 | 0 | 1 | 1 | 1 | 4 |
|  | 0 | 0 | 0 | 0 | 0 | 1 | 0 | 1 |
| Do combination HIV prevention programmes result in increased empowerment, inclusion and agency to demand equal rights for marginalised populations in 1ow-income and middle-income countries? A systematic review | 1 | 1 | 1 | 0 | 1 | 0 | 0 | 4 |
|  | 1 | 0 | 0 | 1 | 1 | 1 | 1 | 5 |
|  | 0 | 0 | 0 | 0 | 0 | 0 | 0 | 0 |
|  | 1 | 1 | 0 | 0 | 1 | 1 | 0 | 4 |
|  | 0 | 0 | 1 | 1 | 0 | 1 | 0 | 3 |
| Zumba®, Fat Mass and Maximum Oxygen Consumption: A Systematic Review and Meta-Analysis | 1 | 1 | 0 | 0 | 0 | 1 | 1 | 4 |
|  | 1 | 1 | 0 | 0 | 1 | 1 | 1 | 5 |
|  | 1 | 1 | 0 | 0 | 0 | 1 | 1 | 4 |
|  | 0 | 0 | 0 | 0 | 1 | 1 | 1 | 3 |
|  | 0 | 0 | 0 | 0 | 0 | 1 | 1 | 2 |
|  | 0 | 0 | 0 | 0 | 1 | 1 | 1 | 3 |
|  | 0 | 0 | 0 | 0 | 1 | 1 | 1 | 3 |
|  | 1 | 0 | 0 | 0 | 1 | 1 | 1 | 4 |
| Effects of psychosocial interventions on children affected by parental HIV | 1 | 1 | 1 | 1 | 0 | 1 | 1 | 6 |
|  | 1 | 0 | 1 | 1 | 1 | 1 | 1 | 6 |
|  | 1 | 1 | 1 | 1 | 0 | 1 | 1 | 6 |
|  | 1 | 0 | 1 | 1 | 1 | 1 | 1 | 6 |
|  | 0 | 0 | 1 | 1 | 1 | 1 | 0 | 4 |
|  | 1 | 1 | 1 | 1 | 1 | 1 | 0 | 6 |
|  | 0 | 0 | 1 | 1 | 1 | 1 | 0 | 4 |
|  | 0 | 0 | 1 | 1 | 1 | 1 | 1 | 5 |
| The effectiveness of vaccination to prevent the papillomavirus infection: a systematic review and meta-analysis | 1 | 1 | 1 | 1 | 0 | 1 | 0 | 5 |
|  | 0 | 0 | 1 | 1 | 1 | 1 | 0 | 4 |
|  | 0 | 0 | 1 | 1 | 0 | 1 | 0 | 3 |
|  | 0 | 0 | 1 | 1 | 0 | 1 | 0 | 3 |
|  | 0 | 1 | 1 | 1 | 1 | 1 | 0 | 5 |
|  | 1 | 0 | 1 | 1 | 1 | 1 | 0 | 5 |
|  | 1 | 0 | 0 | 1 | 0 | 1 | 0 | 3 |
|  | 1 | 0 | 1 | 1 | 0 | 1 | 0 | 4 |
|  | 0 | 0 | 0 | 1 | 1 | 1 | 0 | 3 |
|  | 0 | 0 | 0 | 1 | 1 | 1 | 0 | 3 |
|  | 1 | 0 | 1 | 1 | 0 | 1 | 0 | 4 |
|  | 0 | 0 | 0 | 1 | 1 | 1 | 0 | 3 |
|  | 0 | 0 | 0 | 1 | 1 | 1 | 0 | 3 |
|  | 1 | 0 | 1 | 1 | 0 | 1 | 0 | 4 |
|  | 0 | 1 | 1 | 1 | 1 | 1 | 0 | 5 |
|  | 0 | 0 | 1 | 1 | 1 | 1 | 0 | 4 |
|  | 0 | 1 | 1 | 1 | 1 | 1 | 0 | 5 |
|  | 0 | 0 | 0 | 1 | 0 | 1 | 0 | 2 |
|  | 0 | 0 | 1 | 1 | 1 | 1 | 0 | 4 |
|  | 1 | 0 | 1 | 1 | 1 | 1 | 0 | 5 |
|  | 1 | 0 | 1 | 1 | 1 | 1 | 0 | 5 |
|  | 1 | 1 | 1 | 1 | 0 | 1 | 0 | 5 |
|  | 1 | 0 | 0 | 1 | 1 | 1 | 0 | 4 |
|  | 1 | 1 | 1 | 1 | 1 | 1 | 0 | 6 |
|  | 1 | 1 | 0 | 1 | 1 | 1 | 0 | 5 |
|  | 0 | 0 | 0 | 0 | 0 | 1 | 0 | 1 |
|  | 1 | 1 | 0 | 1 | 0 | 1 | 0 | 4 |
|  | 1 | 0 | 1 | 1 | 1 | 1 | 0 | 5 |
|  | 1 | 0 | 1 | 1 | 1 | 1 | 0 | 5 |
| Differences In Gastrointestinal Safety Profiles Among Novel Oral Anticoagulants: Evidence From A Network Meta-Analysis | 1 | 1 | 1 | 1 | 1 | 1 | 1 | 7 |
|  | 1 | 1 | 1 | 1 | 1 | 1 | 1 | 7 |
|  | 1 | 1 | 1 | 1 | 1 | 1 | 1 | 7 |
|  | 1 | 1 | 1 | 1 | 1 | 1 | 1 | 7 |
|  | 1 | 1 | 1 | 1 | 1 | 1 | 1 | 7 |
|  | 1 | 1 | 1 | 1 | 1 | 1 | 1 | 7 |
|  | 1 | 1 | 1 | 1 | 1 | 1 | 1 | 7 |
|  | 1 | 1 | 1 | 1 | 1 | 1 | 1 | 7 |
|  | 1 | 1 | 1 | 1 | 1 | 1 | 1 | 7 |
|  | 1 | 1 | 1 | 1 | 1 | 1 | 1 | 7 |
|  | 1 | 1 | 1 | 1 | 1 | 1 | 1 | 7 |
|  | 1 | 0 | 0 | 1 | 1 | 1 | 1 | 5 |
|  | 1 | 1 | 1 | 1 | 1 | 1 | 1 | 7 |
|  | 1 | 1 | 1 | 1 | 1 | 1 | 1 | 7 |
|  | 1 | 1 | 1 | 1 | 1 | 1 | 1 | 7 |
|  | 1 | 1 | 1 | 1 | 1 | 1 | 1 | 7 |
|  | 1 | 1 | 1 | 1 | 1 | 1 | 1 | 7 |
|  | 1 | 1 | 1 | 1 | 1 | 1 | 1 | 7 |
|  | 1 | 1 | 1 | 1 | 1 | 1 | 1 | 7 |
|  | 1 | 1 | 1 | 1 | 1 | 1 | 1 | 7 |
|  | 1 | 1 | 1 | 1 | 1 | 1 | 1 | 7 |
|  | 1 | 1 | 1 | 1 | 1 | 1 | 1 | 7 |
|  | 1 | 1 | 1 | 1 | 1 | 1 | 1 | 7 |
|  | 1 | 1 | 1 | 1 | 1 | 1 | 1 | 7 |
|  | 0 | 0 | 1 | 1 | 1 | 1 | 1 | 5 |
| What are the effects of colorectal cancer screening interventions among Asian Americans? A meta- analysis | 1 | 0 | 1 | 1 | 1 | 1 | 0 | 5 |
|  | 1 | 0 | 0 | 1 | 0 | 1 | 0 | 3 |
|  | 1 | 0 | 1 | 1 | 1 | 1 | 0 | 5 |
|  | 1 | 0 | 1 | 1 | 1 | 1 | 0 | 5 |
|  | 1 | 0 | 1 | 1 | 1 | 1 | 0 | 5 |
|  | 1 | 0 | 1 | 1 | 1 | 1 | 0 | 5 |
|  | 1 | 0 | 1 | 1 | 1 | 1 | 0 | 5 |
|  | 1 | 0 | 1 | 1 | 1 | 1 | 0 | 5 |
|  | 1 | 0 | 0 | 0 | 1 | 1 | 0 | 3 |
|  | 1 | 0 | 1 | 0 | 1 | 1 | 0 | 4 |
|  | 0 | 0 | 0 | 0 | 0 | 1 | 0 | 1 |
| Effectiveness of Non-Pharmacological Interventions for Overweight or Obese Infertile Women: A Systematic Review and Meta-Analysis | 1 | 1 | 0 | 0 | 1 | 1 | 1 | 5 |
|  | 0 | 0 | 0 | 0 | 0 | 1 | 1 | 2 |
|  | 1 | 1 | 0 | 0 | 0 | 1 | 0 | 3 |
|  | 1 | 1 | 0 | 1 | 1 | 1 | 1 | 6 |
|  | 1 | 0 | 0 | 1 | 1 | 1 | 1 | 5 |
|  | 1 | 0 | 0 | 0 | 1 | 1 | 1 | 4 |
|  | 1 | 1 | 0 | 0 | 0 | 1 | 0 | 3 |
|  | 0 | 0 | 0 | 0 | 1 | 1 | 1 | 3 |
|  | 1 | 1 | 0 | 0 | 1 | 1 | 1 | 5 |
|  | 1 | 0 | 0 | 0 | 1 | 1 | 1 | 4 |
|  | 1 | 0 | 0 | 0 | 1 | 1 | 1 | 4 |
|  | 0 | 0 | 1 | 0 | 1 | 1 | 1 | 4 |
|  | 1 | 0 | 0 | 0 | 1 | 1 | 1 | 4 |
|  | 1 | 1 | 0 | 1 | 1 | 1 | 1 | 6 |
|  | 1 | 0 | 0 | 0 | 1 | 1 | 1 | 4 |
|  | 1 | 0 | 0 | 0 | 1 | 1 | 1 | 4 |
|  | 0 | 0 | 0 | 1 | 0 | 1 | 0 | 2 |
|  | 1 | 1 | 0 | 1 | 1 | 1 | 1 | 6 |
|  | 1 | 1 | 1 | 1 | 1 | 1 | 1 | 7 |
|  | 1 | 0 | 0 | 0 | 0 | 0 | 1 | 2 |
|  | 0 | 0 | 0 | 0 | 1 | 1 | 1 | 3 |
| The association between balance and free-living physical activity in an older community-dwelling adult population: a systematic review and meta-analysis | 0 | 0 | 0 | 0 | 0 | 1 | 0 | 1 |
|  | 0 | 0 | 0 | 0 | 1 | 1 | 0 | 2 |
|  | 1 | 0 | 0 | 1 | 1 | 0 | 0 | 3 |
|  | 0 | 1 | 0 | 1 | 0 | 1 | 0 | 3 |
| The Efficacy of Workplace Interventions on Improving the Dietary, Physical Activity and Sleep Behaviours of School and Childcare Staff: A Systematic Review | 0 | 0 | 0 | 0 | 1 | 0 | 0 | 1 |
|  | 0 | 0 | 0 | 0 | 0 | 0 | 0 | 0 |
|  | 0 | 0 | 0 | 0 | 1 | 0 | 1 | 2 |
| Physical Activity Interventions in Faith-Based Organizations: A Systematic Review | 0 | 0 | 0 | 0 | 0 | 0 | 0 | 0 |
|  | 0 | 0 | 1 | 0 | 0 | 1 | 0 | 2 |
|  | 0 | 0 | 1 | 0 | 0 | 0 | 1 | 2 |
|  | 0 | 0 | 1 | 0 | 0 | 0 | 0 | 1 |
|  | 0 | 0 | 1 | 0 | 0 | 0 | 1 | 2 |
|  | 0 | 0 | 1 | 0 | 0 | 0 | 0 | 1 |
|  | 0 | 0 | 1 | 0 | 0 | 0 | 0 | 1 |
|  | 1 | 0 | 0 | 0 | 0 | 1 | 0 | 2 |
|  | 1 | 0 | 1 | 0 | 1 | 1 | 0 | 4 |
|  | 0 | 0 | 1 | 0 | 1 | 0 | 0 | 2 |
|  | 0 | 0 | 1 | 0 | 0 | 0 | 0 | 1 |
|  | 0 | 0 | 1 | 0 | 0 | 0 | 0 | 1 |
|  | 0 | 0 | 1 | 0 | 0 | 1 | 1 | 3 |
|  | 1 | 0 | 1 | 0 | 0 | 0 | 1 | 3 |
|  | 0 | 0 | 1 | 1 | 0 | 0 | 0 | 2 |
|  | 0 | 0 | 1 | 0 | 0 | 1 | 0 | 2 |
|  | 0 | 0 | 1 | 1 | 0 | 0 | 0 | 2 |
| The impact of user charges on health outcomes in low-income and middle-income countries: a systematic review | 1 | 1 | 0 | 1 | 1 | 1 | 0 | 5 |
| Using pictures to convey health information: A systematic review and meta-analysis of the effects on patient and consumer health behaviors and outcomes | 0 | 0 | 0 | 1 | 0 | 0 | 0 | 1 |
|  | 1 | 0 | 0 | 1 | 0 | 0 | 0 | 2 |
|  | 0 | 0 | 0 | 0 | 0 | 0 | 0 | 0 |
|  | 0 | 0 | 0 | 1 | 0 | 0 | 0 | 1 |
|  | 1 | 1 | 1 | 1 | 0 | 0 | 0 | 4 |
|  | 0 | 1 | 0 | 0 | 0 | 0 | 0 | 1 |
|  | 1 | 1 | 1 | 0 | 0 | 0 | 0 | 3 |
|  | 0 | 1 | 1 | 1 | 0 | 0 | 0 | 3 |
|  | 1 | 1 | 0 | 0 | 0 | 0 | 0 | 2 |
|  | 1 | 0 | 0 | 0 | 0 | 0 | 0 | 1 |
|  | 1 | 1 | 0 | 1 | 0 | 0 | 0 | 3 |
|  | 0 | 0 | 0 | 0 | 0 | 0 | 0 | 0 |
|  | 1 | 1 | 0 | 1 | 0 | 0 | 0 | 3 |
|  | 0 | 0 | 0 | 0 | 0 | 0 | 0 | 0 |
|  | 1 | 0 | 0 | 0 | 0 | 0 | 0 | 1 |
|  | 1 | 1 | 1 | 0 | 0 | 0 | 0 | 3 |
|  | 0 | 0 | 0 | 0 | 0 | 0 | 0 | 0 |
|  | 0 | 0 | 0 | 0 | 0 | 0 | 0 | 0 |
|  | 1 | 1 | 0 | 0 | 0 | 0 | 0 | 2 |
|  | 1 | 0 | 0 | 0 | 0 | 0 | 0 | 1 |
|  | 1 | 0 | 1 | 1 | 0 | 0 | 0 | 3 |
|  | 0 | 0 | 0 | 1 | 0 | 0 | 1 | 2 |
|  | 0 | 0 | 0 | 0 | 0 | 0 | 0 | 0 |
|  | 0 | 1 | 1 | 1 | 0 | 0 | 0 | 3 |
|  | 0 | 0 | 0 | 0 | 0 | 0 | 0 | 0 |
|  | 1 | 0 | 0 | 0 | 0 | 0 | 0 | 1 |
|  | 0 | 0 | 0 | 0 | 0 | 0 | 1 | 1 |
|  | 0 | 0 | 0 | 0 | 0 | 0 | 0 | 0 |
|  | 1 | 1 | 0 | 1 | 0 | 0 | 0 | 3 |
|  | 0 | 0 | 0 | 0 | 0 | 0 | 0 | 0 |
|  | 0 | 0 | 0 | 0 | 0 | 0 | 0 | 0 |
|  | 1 | 0 | 0 | 1 | 1 | 0 | 1 | 4 |
|  | 1 | 0 | 0 | 0 | 1 | 0 | 1 | 3 |
|  | 0 | 0 | 0 | 0 | 0 | 0 | 0 | 0 |
|  | 1 | 1 | 0 | 1 | 1 | 0 | 0 | 4 |
|  | 0 | 0 | 0 | 0 | 0 | 0 | 0 | 0 |
|  | 1 | 1 | 0 | 0 | 1 | 1 | 0 | 4 |
|  | 0 | 0 | 0 | 0 | 0 | 0 | 0 | 0 |
|  | 0 | 0 | 0 | 0 | 1 | 0 | 1 | 2 |
|  | 0 | 0 | 0 | 0 | 1 | 0 | 0 | 1 |
|  | 0 | 0 | 0 | 0 | 0 | 0 | 0 | 0 |
|  | 1 | 1 | 0 | 1 | 1 | 0 | 1 | 5 |
|  | 1 | 1 | 0 | 1 | 1 | 1 | 1 | 6 |
|  | 1 | 0 | 0 | 0 | 0 | 0 | 0 | 1 |
|  | 1 | 1 | 0 | 1 | 1 | 0 | 0 | 4 |
|  | 1 | 0 | 0 | 1 | 1 | 0 | 0 | 3 |
|  | 1 | 0 | 0 | 0 | 1 | 0 | 0 | 2 |
|  | 1 | 0 | 0 | 0 | 1 | 0 | 0 | 2 |
|  | 1 | 0 | 0 | 0 | 1 | 0 | 0 | 2 |
|  | 1 | 0 | 0 | 0 | 0 | 0 | 0 | 1 |
|  | 1 | 0 | 0 | 0 | 1 | 1 | 1 | 4 |
|  | 1 | 0 | 0 | 1 | 1 | 1 | 0 | 4 |
|  | 1 | 0 | 0 | 0 | 1 | 0 | 0 | 2 |
|  | 1 | 0 | 0 | 0 | 1 | 1 | 0 | 3 |
| **Total score** | **184** | **113** | **101** | **135** | **164** | **176** | **118** | **M (4)** |

Legend: Each row represents a primary study that was included in the systematic review. Each cell is either “1” when the answer to the corresponding question was “yes” or “partial yes” or “0” if the answer was “no” or “cannot tell”.

It is worth noting that items were analyzed based on the assessment of the authors of the included reviews. Based on the detailed item score of the used assessment tool of the included reviews, we re-analyzed the methodological quality of primary studies of the included reviews.
