# Supplementary material for: Characteristics of the sources, evaluation, and grading of the certainty of evidence in systematic reviews in public health: A methodological study
Source: Front Public Health. 2023 Mar 30;11:998588. doi: 10.3389/fpubh.2023.998588 (PMC10097925; doi:10.3389/fpubh.2023.998588)
Supplement: Supplementary file 4 [file Table_4.DOCX]

**Appendix 5** Item scores for the Critical Appraisal Skills Program tool used for qualitative studies

| Title of the systematic review | Clear statement of aims | Qualitative methods justified | Appropriate design for research aims | Appropriate recruitment strategy | Confidence in data collection | Personal biases | Ethical considerations | Confidence in data analysis | Clear statement of findings | Value of research | Total score |
| --- | --- | --- | --- | --- | --- | --- | --- | --- | --- | --- | --- |
| A systematic literature review of reported challenges in health care delivery to migrants and refugees in high-income countries - the 3C model | 1 | 1 | 0 | 1 | 1 | 0 | 1 | 1 | 1 | 1 | 8 |
|  | 1 | 1 | 1 | 1 | 1 | 0 | 1 | 1 | 1 | 1 | 9 |
|  | 1 | 1 | 1 | 1 | 1 | 0 | 1 | 0 | 1 | 1 | 8 |
|  | 1 | 1 | 1 | 0 | 1 | 0 | 1 | 1 | 1 | 1 | 8 |
|  | 1 | 1 | 1 | 1 | 1 | 0 | 1 | 0 | 1 | 1 | 8 |
|  | 1 | 1 | 1 | 1 | 1 | 0 | 1 | 1 | 1 | 1 | 9 |
|  | 1 | 1 | 1 | 1 | 1 | 0 | 1 | 0 | 1 | 1 | 8 |
|  | 1 | 1 | 1 | 1 | 1 | 0 | 1 | 1 | 1 | 1 | 9 |
|  | 1 | 1 | 0 | 0 | 0 | 0 | 0 | 0 | 1 | 1 | 4 |
|  | 1 | 1 | 1 | 1 | 1 | 0 | 1 | 1 | 1 | 1 | 9 |
|  | 1 | 1 | 1 | 1 | 1 | 0 | 1 | 1 | 1 | 1 | 9 |
|  | 1 | 1 | 1 | 1 | 1 | 0 | 1 | 1 | 1 | 1 | 9 |
|  | 1 | 1 | 1 | 1 | 1 | 0 | 1 | 1 | 1 | 1 | 9 |
|  | 1 | 1 | 1 | 1 | 1 | 1 | 1 | 1 | 1 | 1 | 10 |
|  | 1 | 1 | 1 | 1 | 1 | 1 | 1 | 1 | 1 | 1 | 10 |
|  | 1 | 1 | 1 | 1 | 0 | 0 | 1 | 1 | 1 | 1 | 8 |
|  | 1 | 1 | 1 | 1 | 1 | 1 | 1 | 0 | 1 | 1 | 9 |
| Meta-synthesis of Qualitative Research of Pre-exposure Prophylaxis (PrEP) Adherence Among Men Who Have Sex With Men (MSM) | 1 | 1 | 1 | 1 | 1 | 1 | 1 | 1 | 1 | 1 | 10 |
|  | 1 | 1 | 1 | 1 | 1 | 1 | 1 | 1 | 1 | 1 | 10 |
|  | 1 | 1 | 1 | 1 | 1 | 1 | 1 | 1 | 1 | 1 | 10 |
|  | 1 | 1 | 1 | 1 | 1 | 1 | 1 | 1 | 1 | 1 | 10 |
|  | 1 | 1 | 1 | 1 | 1 | 1 | 1 | 1 | 1 | 1 | 10 |
|  | 1 | 1 | 1 | 1 | 1 | 1 | 1 | 1 | 1 | 1 | 10 |
| Cost-related medication nonadherence in Canada: a systematic review of prevalence, predictors, and clinical impact | 1 | 1 | 1 | 1 | 1 | 1 | 1 | 1 | 1 | 1 | 10 |
|  | 1 | 1 | 1 | 0 | 1 | 0 | 1 | 1 | 1 | 1 | 8 |
| Social participation of people with chronic wounds: A systematic review | 1 | 1 | 1 | 1 | 1 | 0 | 1 | 0 | 1 | 1 | 8 |
|  | 1 | 1 | 0 | 1 | 1 | 0 | 1 | 1 | 1 | 1 | 8 |
|  | 0 | 1 | 1 | 1 | 0 | 0 | 1 | 1 | 1 | 0 | 6 |
|  | 1 | 1 | 1 | 0 | 1 | 1 | 0 | 1 | 1 | 0 | 7 |
|  | 1 | 1 | 1 | 1 | 1 | 0 | 1 | 0 | 0 | 0 | 6 |
|  | 0 | 0 | 0 | 0 | 0 | 0 | 0 | 0 | 1 | 0 | 1 |
|  | 1 | 1 | 0 | 1 | 0 | 0 | 1 | 0 | 1 | 0 | 5 |
|  | 1 | 1 | 0 | 1 | 0 | 0 | 1 | 0 | 1 | 0 | 5 |
|  | 1 | 1 | 1 | 1 | 1 | 0 | 1 | 1 | 1 | 1 | 9 |
|  | 1 | 1 | 1 | 1 | 1 | 0 | 1 | 1 | 1 | 1 | 9 |
|  | 0 | 1 | 0 | 0 | 0 | 1 | 1 | 0 | 0 | 0 | 3 |
|  | 1 | 1 | 1 | 1 | 1 | 0 | 1 | 1 | 0 | 0 | 7 |
|  | 1 | 1 | 1 | 1 | 0 | 0 | 1 | 1 | 1 | 0 | 7 |
|  | 1 | 1 | 0 | 0 | 0 | 0 | 1 | 1 | 1 | 0 | 5 |
|  | 0 | 1 | 0 | 1 | 1 | 0 | 0 | 1 | 1 | 0 | 5 |
|  | 0 | 1 | 0 | 1 | 1 | 0 | 1 | 1 | 1 | 1 | 7 |
|  | 1 | 1 | 0 | 0 | 0 | 0 | 1 | 0 | 1 | 1 | 5 |
|  | 1 | 1 | 1 | 0 | 1 | 1 | 1 | 1 | 1 | 1 | 9 |
|  | 1 | 1 | 1 | 1 | 0 | 0 | 0 | 1 | 0 | 0 | 5 |
|  | 1 | 1 | 1 | 0 | 0 | 1 | 0 | 1 | 1 | 0 | 6 |
|  | 1 | 1 | 1 | 1 | 1 | 0 | 1 | 1 | 0 | 1 | 8 |
|  | 1 | 1 | 0 | 1 | 1 | 0 | 1 | 1 | 1 | 1 | 8 |
|  | 0 | 1 | 0 | 1 | 0 | 0 | 0 | 0 | 0 | 0 | 2 |
|  | 1 | 1 | 0 | 0 | 0 | 0 | 0 | 1 | 0 | 0 | 3 |
| Coping with Migration-Related Stressors: A Systematic Review of the Literature | 1 | 1 | 1 | 1 | 1 | 0 | 0 | 1 | 1 | 1 | 8 |
|  | 1 | 1 | 1 | 1 | 1 | 0 | 1 | 1 | 1 | 1 | 9 |
|  | 1 | 1 | 1 | 1 | 1 | 0 | 0 | 1 | 1 | 1 | 8 |
|  | 1 | 1 | 1 | 1 | 1 | 0 | 1 | 1 | 1 | 1 | 9 |
|  | 1 | 1 | 1 | 1 | 1 | 0 | 0 | 1 | 1 | 1 | 8 |
|  | 1 | 1 | 1 | 1 | 1 | 0 | 0 | 1 | 1 | 1 | 8 |
|  | 1 | 1 | 1 | 0 | 1 | 0 | 0 | 1 | 1 | 1 | 7 |
|  | 1 | 1 | 1 | 0 | 1 | 0 | 0 | 1 | 1 | 1 | 7 |
|  | 1 | 1 | 1 | 1 | 1 | 0 | 0 | 1 | 1 | 1 | 8 |
|  | 1 | 1 | 1 | 1 | 1 | 0 | 1 | 1 | 1 | 1 | 9 |
|  | 1 | 1 | 1 | 0 | 0 | 0 | 0 | 1 | 1 | 1 | 6 |
|  | 1 | 1 | 1 | 1 | 1 | 0 | 1 | 1 | 1 | 1 | 9 |
|  | 1 | 1 | 1 | 1 | 1 | 0 | 0 | 1 | 1 | 1 | 8 |
|  | 1 | 1 | 0 | 0 | 0 | 0 | 0 | 0 | 0 | 1 | 3 |
|  | 1 | 1 | 1 | 1 | 1 | 0 | 0 | 1 | 1 | 1 | 8 |
|  | 1 | 1 | 1 | 1 | 1 | 0 | 1 | 1 | 1 | 1 | 9 |
|  | 1 | 1 | 1 | 1 | 1 | 1 | 1 | 1 | 1 | 1 | 10 |
|  | 1 | 1 | 1 | 1 | 1 | 0 | 0 | 1 | 1 | 1 | 8 |
|  | 1 | 1 | 1 | 1 | 1 | 0 | 1 | 1 | 1 | 1 | 9 |
|  | 1 | 1 | 1 | 1 | 1 | 1 | 0 | 0 | 1 | 1 | 8 |
|  | 1 | 1 | 1 | 1 | 1 | 0 | 0 | 1 | 1 | 1 | 8 |
|  | 1 | 1 | 1 | 1 | 1 | 0 | 0 | 1 | 1 | 1 | 8 |
|  | 1 | 1 | 1 | 1 | 1 | 0 | 0 | 1 | 1 | 1 | 8 |
|  | 1 | 1 | 1 | 0 | 1 | 1 | 0 | 1 | 1 | 1 | 8 |
|  | 1 | 1 | 1 | 1 | 1 | 0 | 0 | 1 | 1 | 1 | 8 |
|  | 1 | 1 | 1 | 1 | 1 | 1 | 1 | 1 | 1 | 1 | 10 |
| Experiences of adults with intellectual disabilities accessing acute hospital services: A systematic review of the international evidence | 1 | 1 | 1 | 1 | 1 | 0 | 1 | 1 | 1 | 1 | 9 |
|  | 1 | 1 | 1 | 0 | 1 | 1 | 0 | 0 | 1 | 1 | 7 |
|  | 1 | 1 | 1 | 1 | 1 | 1 | 1 | 1 | 1 | 1 | 10 |
|  | 1 | 1 | 1 | 1 | 1 | 1 | 1 | 1 | 1 | 1 | 10 |
|  | 1 | 1 | 1 | 1 | 1 | 0 | 0 | 1 | 1 | 1 | 8 |
| Factors Influencing Pregnancy and Postpartum Weight Management in Women of African and Caribbean Ancestry Living in High Income Countries: Systematic Review and Evidence Synthesis Using a Behavioral Change Theoretical Model | 1 | 1 | 1 | 1 | 1 | 1 | 1 | 1 | 1 | 1 | 10 |
|  | 1 | 1 | 1 | 1 | 1 | 1 | 1 | 1 | 1 | 1 | 10 |
|  | 1 | 1 | 0 | 1 | 1 | 0 | 1 | 0 | 0 | 1 | 6 |
|  | 1 | 1 | 1 | 1 | 1 | 1 | 1 | 1 | 1 | 1 | 10 |
|  | 1 | 1 | 1 | 1 | 1 | 1 | 1 | 1 | 1 | 1 | 10 |
|  | 1 | 1 | 1 | 1 | 1 | 1 | 1 | 1 | 1 | 1 | 10 |
|  | 1 | 1 | 1 | 1 | 1 | 1 | 1 | 1 | 1 | 1 | 10 |
|  | 1 | 1 | 1 | 1 | 1 | 1 | 1 | 1 | 1 | 1 | 10 |
|  | 1 | 1 | 1 | 1 | 1 | 1 | 1 | 1 | 1 | 1 | 10 |
|  | 1 | 1 | 1 | 1 | 1 | 1 | 1 | 1 | 1 | 1 | 10 |
|  | 1 | 1 | 1 | 1 | 1 | 1 | 1 | 1 | 1 | 1 | 10 |
|  | 1 | 1 | 1 | 1 | 1 | 1 | 1 | 1 | 1 | 1 | 10 |
|  | 1 | 1 | 1 | 1 | 1 | 1 | 1 | 1 | 1 | 1 | 10 |
|  | 1 | 1 | 1 | 1 | 1 | 1 | 1 | 1 | 1 | 1 | 10 |
|  | 1 | 1 | 1 | 1 | 1 | 1 | 1 | 1 | 1 | 1 | 10 |
|  | 1 | 1 | 1 | 1 | 1 | 0 | 1 | 1 | 1 | 1 | 9 |
|  | 1 | 1 | 1 | 1 | 1 | 1 | 1 | 1 | 1 | 1 | 10 |
|  | 1 | 1 | 1 | 1 | 1 | 1 | 1 | 1 | 1 | 1 | 10 |
|  | 1 | 1 | 1 | 1 | 1 | 1 | 1 | 1 | 1 | 1 | 10 |
|  | 1 | 1 | 1 | 1 | 1 | 1 | 1 | 1 | 1 | 1 | 10 |
|  | 1 | 1 | 1 | 1 | 1 | 1 | 1 | 1 | 1 | 1 | 10 |
|  | 1 | 1 | 1 | 1 | 1 | 1 | 1 | 1 | 1 | 1 | 10 |
|  | 1 | 1 | 1 | 1 | 1 | 1 | 1 | 1 | 1 | 1 | 10 |
|  | 1 | 1 | 1 | 1 | 1 | 1 | 1 | 1 | 1 | 1 | 10 |
| **Total score** | **98** | **103** | **88** | **87** | **88** | **43** | **76** | **87** | **95** | **89** | **M (9)** |

Legend: Each row represents a primary study that was included in the systematic review. Each cell is either “1” when the answer to the corresponding question was “yes” or “partial yes” or “0” if the answer was “no” or “cannot tell”.

It is worth noting that items were analyzed based on the assessment of the authors of the included reviews. Based on the detailed item score of the used assessment tool of the included reviews, we re-analyzed the methodological quality of primary studies of the included reviews.
