# Supplementary material for: Characteristics of the sources, evaluation, and grading of the certainty of evidence in systematic reviews in public health: A methodological study
Source: Front Public Health. 2023 Mar 30;11:998588. doi: 10.3389/fpubh.2023.998588 (PMC10097925; doi:10.3389/fpubh.2023.998588)
Supplement: Supplementary file 5 [file Data_Sheet_1.DOCX]

**Appendix 1. Search Strategy**

**Medline (via PubMed)**

1. “Meta-analysis” [Title]
2. “Meta-Analyses” [Title]
3. “meta-synthesis” [Title]
4. “meta-regression” [Title]
5. “gathering analysis” [Title]
6. “systematic review” [Title]
7. “Systematic evaluation” [Title]
8. “Systematic assessment” [Title]
9. "systematic literature reviews"[Title]
10. "systematic literature review"[Title]
11. “Systematic reviews” [Title]
12. “System evaluation” [Title]
13. “Systematical reviews” [Title]
14. “System Assessment” [Title]
15. “Systemic review” [Title]
16. “Systemic reviews” [Title]
17. “integrative research review” [Title]
18. “integrative review” [Title]
19. “research synthesis” [Title]
20. “research integration” [Title]
21. “data synthesis” [Title]
22. “evaluation of system” [Title]
23. “systematic scoping review” [Title]
24. “systematic scoping reviews” [Title]
25. “systematic narrative review” [Title]
26. “systematic narrative reviews” [Title]
27. “systematic qualitative review” [Title]
28. “systematic qualitative reviews” [Title]
29. “systematic evidence review” [Title]
30. “systematic evidence reviews” [Title]
31. “systematic quantitative review” [Title]
32. “systematic quantitative reviews” [Title]
33. “systematic meta-review” [Title]
34. “systematic meta-reviews” [Title]
35. “systematic critical review” [Title]
36. “systematic critical reviews” [Title]
37. “systematic mixed studies review” [Title]
38. “systematic mixed studies reviews” [Title]
39. “systematic mapping review” [Title]
40. “systematic mapping reviews” [Title]
41. “systematic cochrane review” [Title]
42. “systematic cochrane reviews” [Title]
43. “systematic integrative review” [Title]
44. “systematic integrative reviews” [Title]
45. #1-44/ OR
46. Journal Name [Journal]
47. #45 AND #46
48. lim 2018/1/1-2021/4/1
